# Two NHX‐type transporters from *Helianthus tuberosus* improve the tolerance of rice to salinity and nutrient deficiency stress

**DOI:** 10.1111/pbi.12773

**Published:** 2017-09-21

**Authors:** Yang Zeng, Qing Li, Haiya Wang, Jianliang Zhang, Jia Du, Huimin Feng, Eduardo Blumwald, Ling Yu, Guohua Xu

**Affiliations:** ^1^ State Key Laboratory of Crop Genetics and Germplasm Enhancement Nanjing Agricultural University Nanjing China; ^2^ Key Laboratory of Plant Nutrition and Fertilization in Low‐Middle Reaches of the Yangtze River Ministry of Agriculture Nanjing Agricultural University Nanjing China; ^3^ Department of Plant Sciences University of California Davis CA USA

**Keywords:** *Helianthus tuberosus*, NHX‐type transporters, nutrient deficiency, potassium, rice, salt stress

## Abstract

The NHX‐type cation/H^+^ transporters in plants have been shown to mediate Na^+^(K^+^)/H^+^ exchange for salinity tolerance and K^+^ homoeostasis. In this study, we identified and characterized two *NHX* homologues, *HtNHX1* and *HtNHX2* from an infertile and salinity tolerant species *Helianthus tuberosus* (cv. Nanyu No. 1). *HtNHX1* and *HtNHX2* share identical 5′‐ and 3′‐UTR and coding regions, except for a 342‐bp segment encoding 114 amino acids (L_272_ to Q_385_) which is absent in *HtNHX2*. Both hydroponics and soil culture experiments showed that the expression of *HtNHX1* or *HtNHX2* improved the rice tolerance to salinity. Expression of *HtNHX2*, but not *HtNHX1*, increased rice grain yield, harvest index, total nutrient uptake under K^+^‐limited salt‐stress or general nutrient deficiency conditions. The results provide a novel insight into NHX function in plant mineral nutrition.

## Introduction

Naturally occurred and fertilization‐induced soil salinity, and low nutrient use efficiency are significant constraints in modern agriculture production (Heuer *et al*., 2016; Horie *et al*., 2009; Munns and Gilliham, 2015; Munns and Tester, 2008; Xu *et al*., 2012). At the cellular level, the extrusion of Na^+^ ions at the cell plasma membrane and the compartmentation of Na^+^ into vacuoles are efficient mechanisms to avoid excessive cytosolic Na^+^ concentration and maintain an adequate cytosolic K^+^/Na^+^ ratio (Julkowska and Testerink, 2015; Munns and Tester, 2008). The plasma membrane electrical potential difference of root cells is usually maintained around −120 mV, while the tonoplast (vacuole membrane) potential is positive and around 20–40 mV (Shabala *et al*., 2016). These potentials allow the root to acquire sufficient K^+^ via high‐affinity transporter systems (HATS) in K^+^‐limited soils or via low‐affinity transporter systems (LATS) at normal external K^+^ supplies. The presence of high external Na^+^ concentrations suppresses the K^+^ conductance through LATS (Qi and Spalding, 2004) and competes with K^+^ uptake through HATS (Alemán *et al*., 2011; Leidi *et al*., 2010), causing a decrease in intracellular K^+^ with a concomitant [K^+^]/[Na^+^] imbalance.

The plant vacuolar Na^+^/H^+^ antiporters (NHXs) were shown originally to mediate the electroneutral Na^+^/H^+^ exchange, driving the excess cytosolic Na^+^ into the vacuole (Apse *et al*., 1999; Blumwald and Poole, 1985; Gaxiola *et al*., 1999). The NHX proteins belong to the large mono‐valent cation/proton transporters (CPA) family, showing three distinct functional clades (Chanroj *et al*., 2012). In Arabidopsis, in addition to the plasma membrane‐located NHX7 and NHX8, also known as SOS (salt‐over‐sensitive), intracellular NHXs sharing high sequence similarity are further divided into type‐I and type‐II, based on their subcellular location (Chanroj *et al*., 2012). Type‐I NHXs (AtNHX1‐AtNHX4) are vacuolar‐located, while type‐II NHXs (AtNHX5 and AtNHX6) are found at endosome, Trans‐Golgi Network (TGN)/Golgi and prevacuolar compartments (Andrés *et al*., 2014; Barragán *et al*., 2012; Bassil *et al*., 2011a,b; McCubbin *et al*., 2014). In the rice genome, there are at least five NHX members with OsNHX1‐OsNHX4 belonging to the type‐I and OsNHX5 to the type‐II (Fukuda *et al*., 2011). The operation of the NHXs also affects the pH gradients across the different luminal compartments (Bassil *et al*., 2012).

Both type‐I and type‐II NHXs have different subcellular localization; they may have different mechanisms in salt tolerance, and other not yet defined functions. Large efforts have been paid to increasing the plant salt tolerance by overexpressing *NHX* orthologue genes from different species. In rice, overexpression of the *NHX1* homologue genes from *Oryza sativa*,* Chenopodium glaucum* and *Atriplex dimorphostegia* resulted in the enhanced tolerance to salt stress (Li *et al*., 2008). However, these NHX proteins actually mediate both Na^+^/H^+^ and K^+^/H^+^ exchange and their functions cannot be solely explained by accumulating Na^+^ into vacuole (Andrés *et al*., 2014; Barragán *et al*., 2012; Bassil *et al*., 2011b; Jiang *et al*., 2010; Leidi *et al*., 2010; Reguera *et al*., 2014).

The type‐II NHXs have also been shown to be involved in salt tolerance. AtNHX5 and AtNHX6 are the only two type‐II NHXs in Arabidopsis. Although their function appears to be redundant, the double knockout *Atnhx5nhx6* displayed high sensitivity to salt stress (Bassil *et al*., 2011a). In tomato, the overexpression of LeNHX2, a type‐II NHX located in prevacuolar compartments and Golgi, enhanced salt tolerance at high external K^+^ levels (Venema *et al*., 2003).

Plants use a number of strategies to deal with high salinity (Munns and Tester, 2008). Halophytes rely heavily on the homoeostasis of three major inorganic ions (Na^+^, Cl^−^, and K^+^) to maintain their osmotic and turgor pressure under saline conditions, while glycophytes predominantly increase the synthesis of compatible solutes (Deinlein *et al*., 2014). For salt tolerant plants grown in high external saline conditions, the efficient compartmentation of Na^+^ into vacuole and other cell organs via intracellular NHXs is of importance, together with the extrusion of Na^+^ at the roots, via plasma membrane‐bound antiporters such as SOS (Deinlein *et al*., 2014; Munns and Tester, 2008).

Jerusalem artichoke (*Helianthus tuberosus* L.) belonging to the same Asteraceae family as sunflower (*Helianthus annuus* L.) is an herbaceous perennial plant that have potential as a biorefinery crop (Johansson *et al*., 2015). *H. tuberosus* is highly tolerant to infertile, drought and saline stresses and its variety Nanyu No. 1 (NY‐1) could fully emerge in the coastal region containing 1% salt at soil surface layer (0–15 cm) and grow well in soil containing 0.5%–0.6% salt or under irrigation with 50%–75% sea water (Long *et al*., 2008). In this study, we isolated two putative *NHX* genes, *HtNHX1* and *HtNHX2,* from *H. tuberosus* cv. NY‐1 and examined their roles in enhancing the tolerance of salt stress and nutrient deficiency in rice, Arabidopsis and yeast. Our results indicate that HtNHX2 could function in enhancing the plant tolerance to salinity stress and improving plant nutrient efficiency.

## Results

### Cloning of salt‐stress up‐regulated *HtNHX1* and *HtNHX2* genes from *Helianthus tuberosus*

To clone *NHX* genes in *H. tuberosus*, we designed the primers (Table S1) based on the conserved regions of known *NHXs* in plants (Figure S1) for PCR amplification of cDNA isolated from the salt‐treated cv. NY‐1. The two different fragments were obtained, and their full lengths were further isolated by 5′ and 3′ RACE‐PCR. The two putative *NHX* genes with 2148‐ and 1806‐bp encoding regions were named *HtNHX1* and *HtNHX2*, respectively.

Interestingly, *HtNHX1* and *HtNHX2* have identical 269 bp of 5′ UTR and 501 bp of 3′UTR. Furthermore, their coding sequences are also identical except a segment of 342 bp in *HtNHX1* encoding 114 amino acids (L_272_ to Q_385_) which is absent in *HtNHX2* (Figure S1A). By alignment of the amino acids sequences of HtNHX1 and HtNHX2 with more than forty NHX members from different plant species, both HtNHX1 and HtNHX2 are classified as IC type‐I NHXs (Figure S1B). Transmembrane topology predicted by TMpred (http://www.ch.embnet.org/software/TMPRED_form.html) or TMHMM2.0 (http://www.cbs.dtu.dk/services/TMHMM/) indicated that HtNHX1 comprised 10 transmembrane domains, while HtNHX2 has seven to eight transmembrane domains (Figure S1CD).

**Figure 1:**
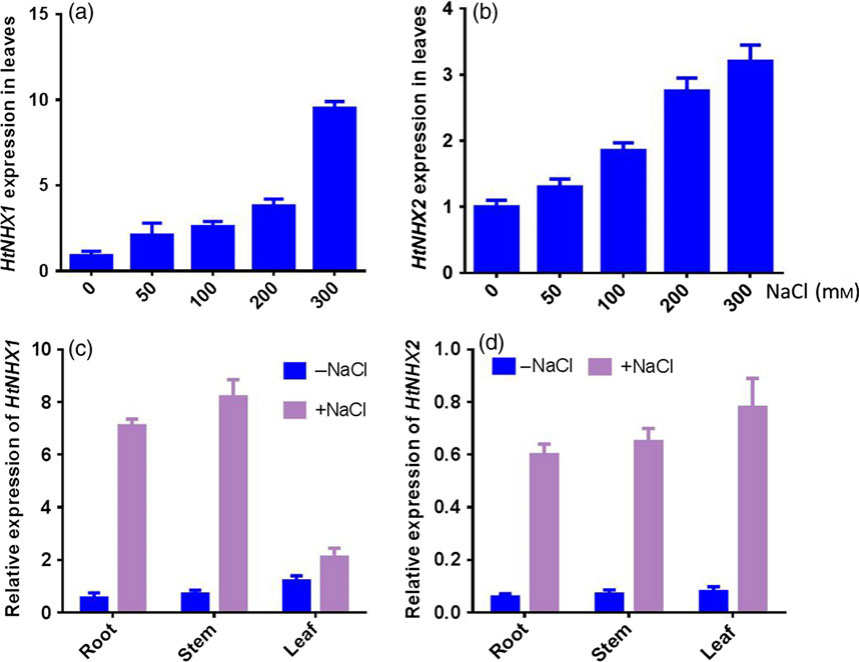
Effects of salt treatments on expression of *HtNHX1* and *HtNHX2* in entire shoot (a, b) and root, stem and leaf (c, d) of *H. tuberosus* grown in nutrient sufficient solution. The seedlings of *H. tuberosus* (cv. Nanyu no. 1) were grown in the nutrient solution for 14 days, and then different amount of NaCl solution was added into the culture solution for 24 h (a, b) and 100 mm NaCl for 12 h (c, d) before the sampling for RNA extraction. The expression of *HtNHX1* and *HtNHX2* was relative to *HtActin* abundance in the cultivar. The bar of each column is SE (standard error) with three biological replicates.

As the expression of most known type‐I NHXs was up‐regulated by salt stress, we analysed the expression of *HtNHX1* and *HtNHX2* in *H. tuberosus* in responses to salt treatments (100 mm NaCl). The expression of *HtNHX1* was determined by real‐time qRT‐PCR with the primers flanking the region absent in *HtNHX2*, while the expression level of *HtNHX2* was distinguished from that of *HtNHX1* by semi‐quantitative RT‐PCR with a product of *HtNHX2* 342 bp shorter than that of *HtNHX1*. As shown in Figure 1a,b, both *HtNHX1* and *HtNHX2* were up‐regulated by increasing the salinity levels. *HtNHX1* expression was enhanced in the roots and stems, and to a lesser extent in leaves, while *HtNHX2* expression was enhanced similarly in all three organs (Figure 1c,d).

### *HtNHX1* and *HtNHX2* expression conferred salt tolerance in rice

**Figure 2:**
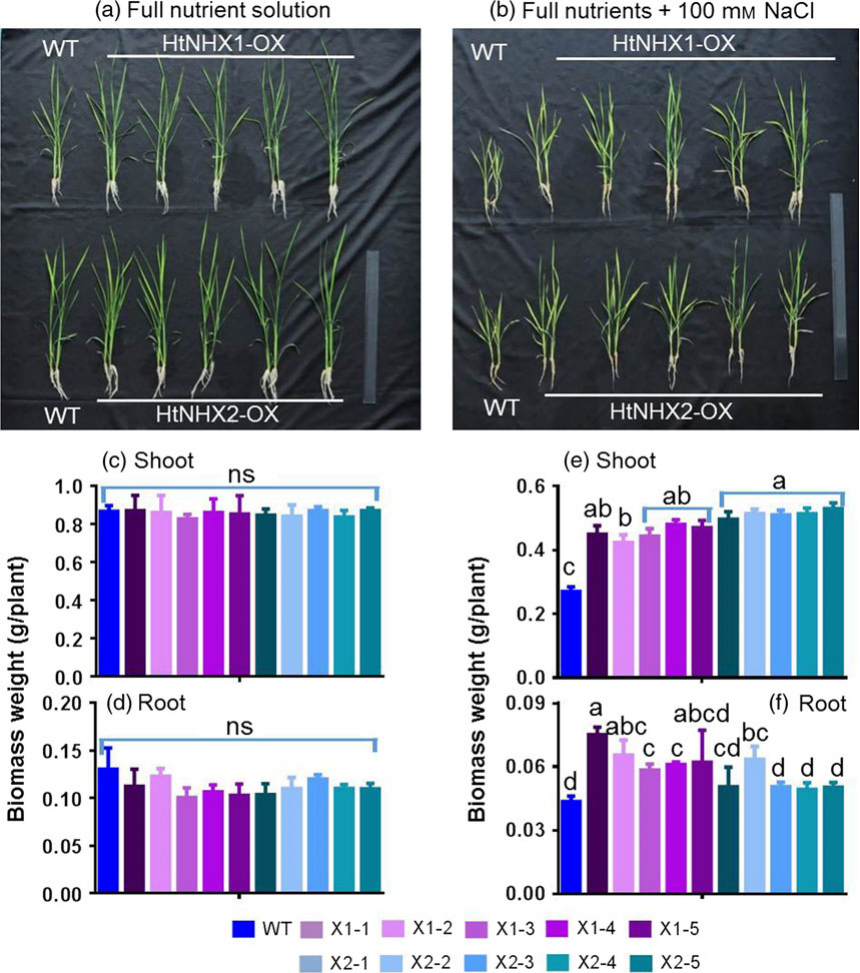
Effects of overexpression of *HtNHX1* and *HtNHX2* on rice growth under different nutrient supply and salt‐stress condition. The rice was grown for 21 days in the solution before the sampling. (a, c, d) IRRI solution with full supply of nutrients (see Materials and methods); (b, e, f) Full nutrient solution + 100 mm NaCl. WT: cv. *Nipponbare*; X1‐1, 1‐2, 1‐3, 1‐4 and 1‐5: five T2 individual transgenic lines of expressing *HtNHX1*; X2‐1, 2‐2, 2‐3, 2‐4 and 2‐5: five T2 individual transgenic lines of expressing *HtNHX2*. Different letter(s) on the column indicates the significant difference at probability at 5%. ns: no significant difference.

**Figure 3:**
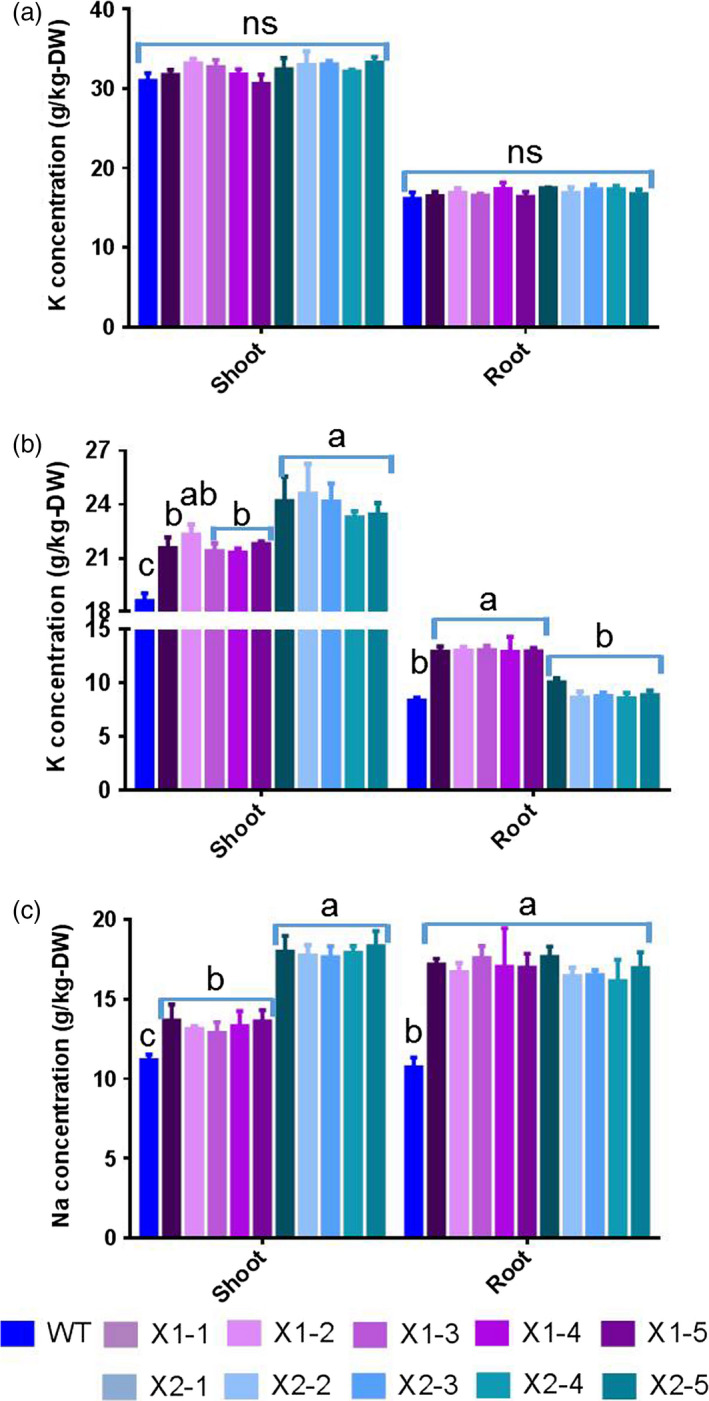
Effects of overexpression of *HtNHX1* and *HtNHX2* on uptake of K^+^ and Na^+^ under full nutrient supply and salt‐stress condition. (a, b) K^+^ concentration in shoot and root under full nutrient supply and nonsalinized condition (1 mm K^+^ + 0 mm Na^+^) (a) and under salinized condition (1 mm K^+^ + 100 mm Na^+^) (b); (c) Na^+^ concentration in shoot and root under salinized condition (1 mm K^+^ + 100 mm NaCl). WT: cv. *Nipponbare*; X1‐1, 1‐2, 1‐3, 1‐4 and 1‐5: five T2 individual transgenic lines of expressing *HtNHX1*; X2‐1, 2‐2, 2‐3, 2‐4 and 2‐5: five T2 individual transgenic lines of expressing *HtNHX2*. Different letter(s) on the column indicates the significant difference at probability at 5%. ns: no significant difference.

To assess HtNHX1 and HtNHX2 functional role(s), *HtNHX1* and *HtNHX2* were overexpressed in rice (cv. Nipponbare) driven by the ubiquitin promoter (see Materials and methods, Figure S2) and salt tolerance tested. Wild‐type (WT) and transgenic lines were grown under normal conditions for 2 weeks, and the seedlings were exposed to 100 mm NaCl for 3 weeks and growth measured (Figure 2). While the expression of *HtNHX1* or *HtNHX2* did not affect plant root or shoot biomass (Figure 2a,c,d), the presence of NaCl induced a reduction of growth in both shoot and roots, but the growth of the transgenic plants was less affected by salt stress (Figure 2b,e,f). When grown under nonsalinized conditions, the K^+^ contents of shoots and roots were similar in WT and transgenic plants, although shoots contained higher K^+^ amounts than roots (Figure 3a). Transgenic plants expressing *HtNHX1* displayed higher shoot and root K^+^ contents (Figure 3b), while transgenic plants expressing *HtNHX2* displayed higher K^+^ contents than WT but similar root K^+^ contents (Figure 3b). All transgenic lines displayed higher shoot and root Na^+^ amounts than WT, although Na^+^ contents were lower in shoots expressing *HtNHX1* than plants expressing *HtNHX2* (Figure 3c).

**Figure 4:**
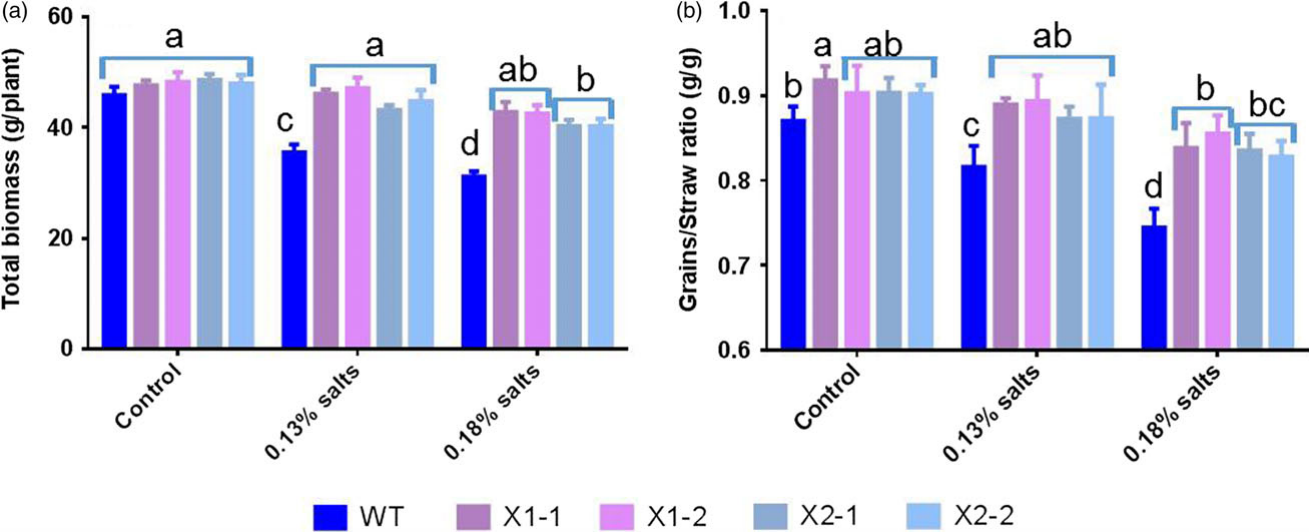
Effects of overexpression of *HtNHX1* and *HtNHX2* on rice growth, harvest index grown in the soil with different amount of additive salts (NaCl). Two‐week‐old seedlings were acclimated to the soil containing 0.1% (1‐g/kg soil) NaCl for 1 week before being transplanted to the pot filled with 40 kg of soil which had been fertilized and mixture with indicated extra amount of salts for 2 weeks. Control: no additive NaCl was provided into the fertile soil (see Table S2); 0.13% and 0.18% salts: adding 1.3 and 1.8 g NaCl per kg soil in the pot. WT: cv. *Nipponbare*; X1‐1, 1‐2: two T2 individual transgenic lines of expressing *HtNHX1*; X2‐1, 2‐2: two T2 individual transgenic lines of expressing *HtNHX2*. Different letter(s) on the column indicates the significant difference at probability at 5%. ns: no significant difference.

We also examined the effects of HtNHX1 and HtNHX2 on grain yields of plants grown under salinized soils (Figure 4). Plant biomass and harvest index (grain/straw ratio) was similar in WT and transgenic plants under control conditions (Figure 4a,b). Under salt treatments, WT plants displayed significant yield penalties, while the yield of the transgenic plants was only slightly affected (Figure 4a,b), confirming the role of HtNHX conferring salt tolerance.

### Roles of HtNHX1 and HtNHX2 in enhancing rice tolerance to K^+^ deficiency and salt tolerance

**Figure 5:**
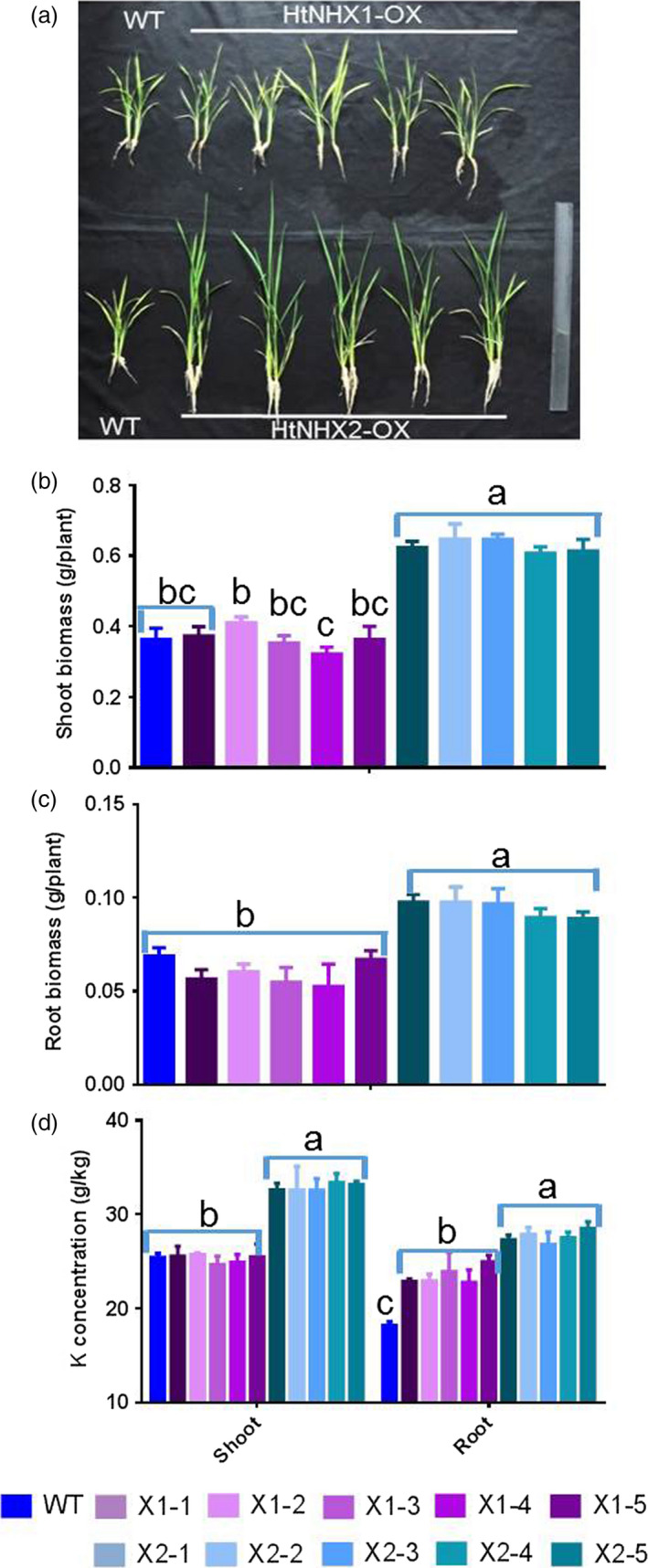
Effects of overexpression of *HtNHX1* and *HtNHX2* on rice growth and K^+^ accumulation under limited K^+^ supply condition. The rice was grown for 21 days in the solution containing 1/4 K^+^ (0.25 mm) of IRRI solution with sufficient supply of all other nutrients (see Materials and methods). (a) Growth phenotypes; (b and c) biomass of shoot (b) and root (c); (d) K^+^ concentration in shoot and root. WT: cv. *Nipponbare*; X1‐1, 1‐2, 1‐3, 1‐4 and 1‐5: five T2 individual transgenic lines of expressing *HtNHX1*; X2‐1, 2‐2, 2‐3, 2‐4 and 2‐5: five T2 individual transgenic lines of expressing *HtNHX2*. Different letter(s) on the column indicates the significant difference at probability at 5%. ns: no significant difference.

When transgenic rice plants were grown under low K^+^ concentrations (i.e. 0.25 mm), the expression of *HtNHX1* did not affect either plant growth or K^+^ contents in the shoot (Figure 5a–d). On the other hand, the overexpression of *HtNHX2* resulted in significant increases in shoot biomass (70%), root biomass (35%) and plant K^+^ contents (25%–55%) (Figure 5a–d).

The functions of HtNHX1 and HtNHX2 in improving rice tolerance to K^+^ deficiency were also tested in low K^+^‐containing soil (Figure S3). When K^+^ fertilizer was applied, there were no differences between WT and transgenic plants expressing *HtNHX1* or *HtNHX2* (Figure S3A, B). However, in low K^+^‐containing soil, the transgenic lines expressing *HtNHX2* displayed about 30% increase in grain yield (Figure S3A), increased harvest index (Figure S3B) and increased straw K^+^ contents (Figure S3C). In contrast, expression of *HtNHX1* significantly decreased rice grain yield and grain to straw ratio by about 40%–50% even though the concentrations of N, P and K^+^ in the straw were not significantly affected (Figure S3A–C).

**Figure 6:**
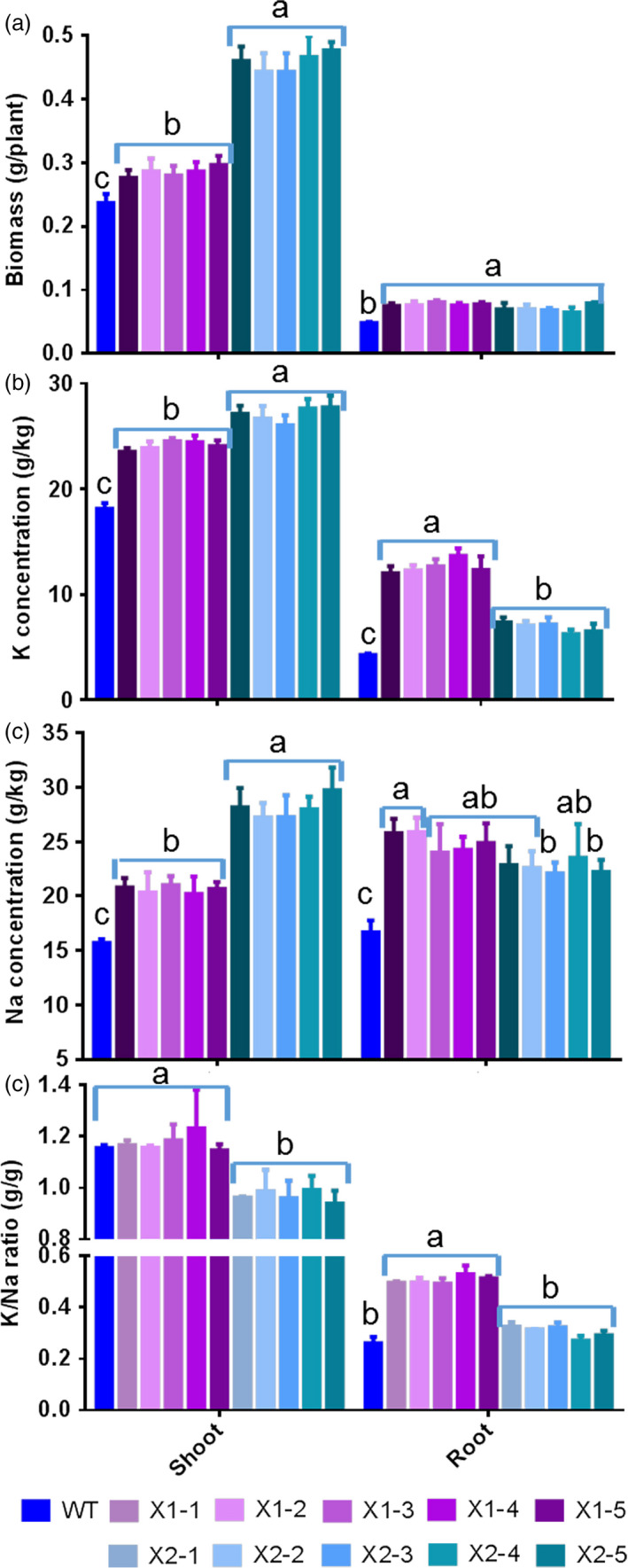
Effects of overexpression of *HtNHX1* and *HtNHX2* on rice biomass production and total K^+^ and Na^+^ accumulation under limited K^+^ supply and salt‐stress condition. The rice was grown for 21 days in the solution containing ¼ K^+^ (0.25 mm) of IRRI solution with sufficient supply of all other nutrients and 100 mm NaCl (see Materials and methods). (a) Biomass of shoot and root; (b, c) concentration of K (b) and Na (c) in shoot and root; (d) K^+^/Na^+^ ratio in shoot and root. WT: cv. *Nipponbare*; X1‐1, 1‐2, 1‐3, 1‐4 and 1‐5: five T2 individual transgenic lines of expressing *HtNHX1*; X2‐1, 2‐2, 2‐3, 2‐4 and 2‐5: five T2 individual transgenic lines of expressing *HtNHX2*. Different letter(s) on the column indicates the significant difference at probability at 5%. ns: no significant difference.

Interestingly, a large difference in the effects of expressing *HtNHX1* or *HtNHX2* in enhancing the tolerance to salt stress under limited K^+^ supply was noted (Figure 6). As compared to WT, the transgenic lines expressing *HtNHX2* showed increased shoot biomass, K^+^ and Na^+^ contents of 95%, 40% and 90%, respectively, while a lesser increase was seen in lines expressing *HtNHX1* (Figure 6a–c). Total shoot K^+^/Na^+^ remained unchanged in plants expressing *HtNHX1,* but decreased significantly in plants expressing *HtNHX2* (Figure 6d). Although comparable increases in root growth and Na^+^ contents were obtained by expressing *HtNHX1* and *HtNHX2*, K^+^ root contents were higher in plants expressing *HtNHX1* than *HtNHX2* (Figure 6a–c), resulting in higher root K^+^/Na^+^ ratio in *HtNHX1* lines (Figure 6d).

### HtNHX2, but not HtNHX1, enhanced rice tolerance to limited supply of major nutrients

**Figure 7:**
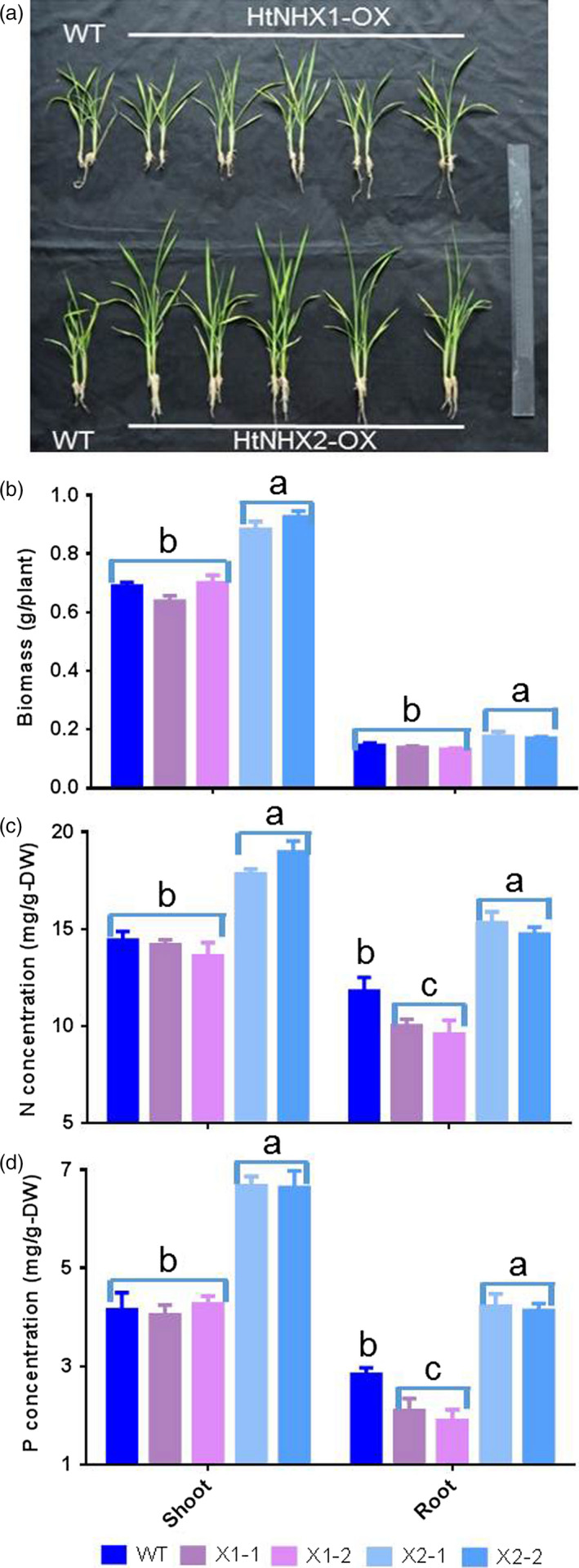
Effects of overexpression of *HtNHX1* and *HtNHX2* on rice growth and N and P concentrations under supply of ¼ concentrations of all the essential nutrients in IRRI culture solution. (a) The rice was grown in IRRI solution with ¼ concentrations of the full supply; (b) biomass of shoot and root; (c, d) concentration of N (c) and P (d) in shoot and root. WT: cv. *Nipponbare*; X1‐1, 1‐2: two T2 individual transgenic lines of expressing *HtNHX1*; X2‐1, 2‐2: two T2 individual transgenic lines of expressing *HtNHX2*. Different letter on the column indicates the significant difference at probability at 5%.

While the ectopic expression of *HtNHX1* or *HtNHX2* did not induce phenotypical differences in rice grown under control conditions, transgenic rice plants expressing *HtNHX2* displayed remarkable higher growth than WT or transgenic plants expressing *HtNHX1*, when grown under low K supply (Figure 5). Considering the essential roles of K^+^ in balancing uptake and distribution of anions, particularly, nitrate and phosphate (Drew and Saker, 1984; Drew *et al*., 1990; Engels and Marschner, 1992) and improving plant growth, we characterized the effects of expressing *HtNHX1* and *HtNHX2* on enhancing the tolerance of rice to nutrient deficiency. The reduction in nutrient supply to 1/4 of its full strength decreased the growth and biomass of WT and *HtNHX1‐*expressing rice equally (Figure 7a,b) while did not affect the growth of *HtNHX2*‐expressing rice plants (Figure 7a,b). The *HtNHX2* lines showed about 35%, 25% and 45% increase in total biomass, N and P contents, respectively, in comparison with WT (Figure 7b–d), indicating that *HtNHX2* expression could remarkably enhance the root acquisition of N and P, thus improving plant growth under limited nutrient supply conditions. In contrast, N and P contents in the *HtNHX1* lines were lower than those in WT. ^15^N quantification analysis of rice grown in low N and K conditions showed that *HtNHX2* expression did not alter root N contents, but significantly increased shoot N contents (Figure S4), suggesting the enhanced N translocation from shoot to root, in addition to the increase in total N uptake.

**Figure 8:**
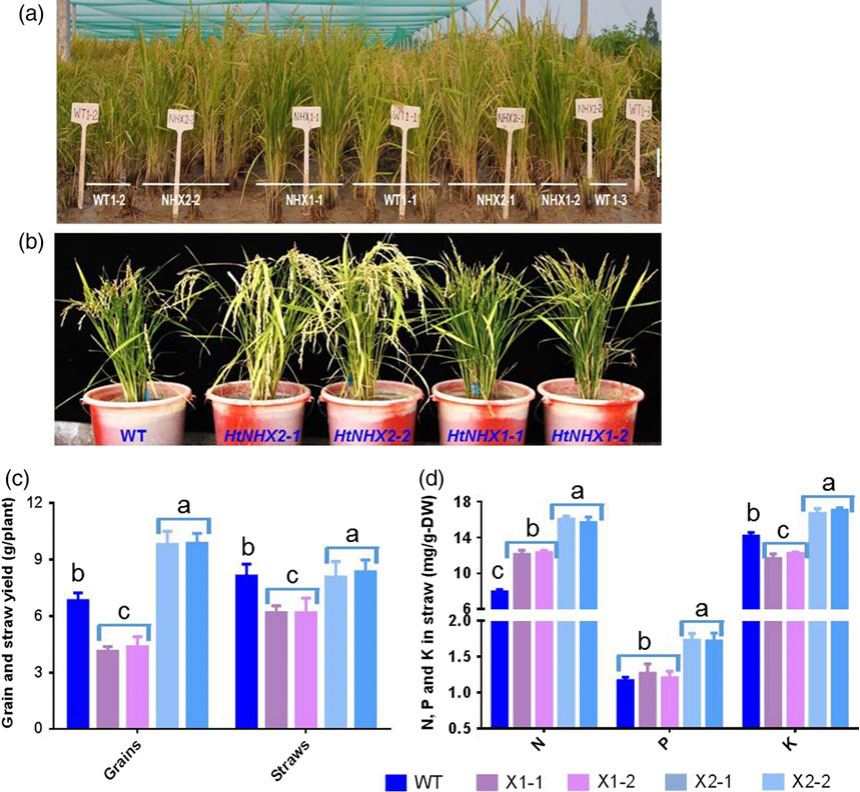
Effects of overexpression of *HtNHX1* and *HtNHX2* on grain yield and straw biomass, and concentrations of N, P, K^+^ in rice grown in nutrient deficient soil. (a, b) The rice was grown in Ledong experimental station, Hainan Province. Soil properties were described in Table S2, and no fertilizer was applied during the season. (c) Grain yield and straw biomass. (d) Concentrations of total N, P and K^+^ in the harvested straws. WT: cv. *Nipponbare*; X1‐1, 1‐2: two T2 individual transgenic lines of expressing *HtNHX1*; X2‐1, 2‐2: two T2 individual transgenic lines of expressing *HtNHX2*. Different letter(s) on the column indicates the significant difference at probability at 5%.

The notion of the role(s) of HtNHX2, but not HtNHX1, in enhancing K^+^ uptake and mediating N and P accumulation was further supported by the growth of rice plants in infertile paddy fields (low N and K contents) (see Table S2). Compared to WT, *HtNHX1* lines showed 20% lower straw weight (biomass) and 40% lower grain yield, resulting in significant lower grain harvest index due to a larger portion of unfilled grains (Figure 8a–c). The *HtNHX1* lines contained significant higher total N, similar P and lower K^+^ in the straw at mature stage (Figure 8d). Remarkably, the *HtNHX2* lines showed 45% increase of total grain yield and 90%, 40% and 13% higher N, P and K^+^ contents in the straw (Figure 8c,d).

### Both HtNHX1 and HtNHX2 could rescue the growth impairment of *nhx5* and *nhx6* double mutant of Arabidopsis

**Figure 9:**
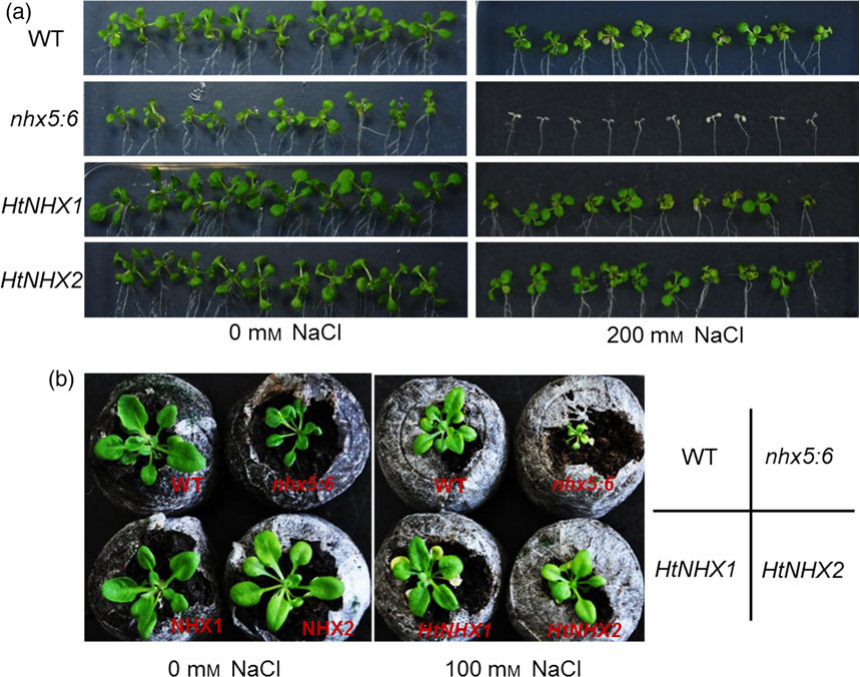
Either *HtNHX1* or *HtNHX2* rescued the growth defect of Arabidopsis *nhx5* and *nhx6* double mutant under both normal and salt‐stress conditions. (a) The growth in agar medium with or without additive NaCl; (b) The growth in the substrate with or without additive NaCl in the pots for 6 weeks.

In Arabidopsis, AtNHX5 and AtNHX6 are located at TGN, Golgi and prevacuolar compartments and the double knockout *Atnhx5nhx6* displayed growth impairment under normal growth condition and high sensitivity to salinity (Bassil *et al*., 2011a). We found that either *HtNHX1* or *HtNHX2* expression could rescue the growth defects and salt sensitivity of the *Atnhx5nhx6* mutant similarly (Figure 9a,b), suggesting a functional similarity between HtNHX1, HtNHX2 and AtNHX5, AtNHX6. Interestingly, only HtNHX1, but not HtNHX2, could enhance the tolerance of *nhx1* mutant yeast (AXT3 strains) to hygromycin (Figure S5). In addition, the transformation of the At*nhx5nhx6* double mutant with *HtNHX1* or *HtNHX2* resulted in increased hygromycin resistance, although HtNHX1‐expressing plants displayed much stronger hygromycin tolerance (Figure S5).

### HtNHX1 and HtNHX2 showed different subcellular localization

**Figure 10:**
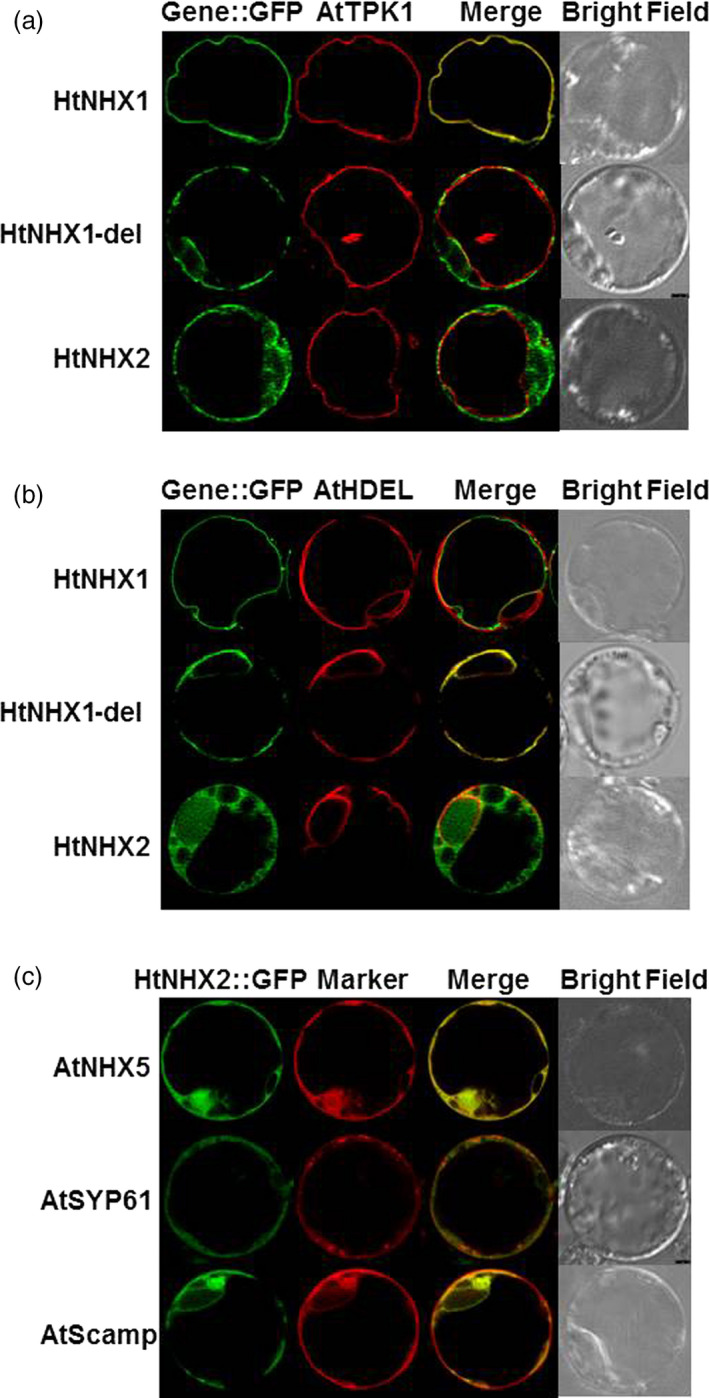
Subcellular localization of HtNHX1 and HtNHX2 in rice protoplast. Expression of HtNHX1, HtNHX2, HtNHX1del fused with GFP, and AtTPK1 (a), AtHDEL (b)?AtNHX5, AtSYP61, AtScamp (c) fused with RFP in rice protoplast cells.. HtNHX1del: expression of mutated HtNHX1 by deletion of conserved eight amino acids in NHX1 group from different plant species (see Figure S1). AtTPK1, AtHDEL, AtNHX5, AtSYP61 and AtScamp are the localization markers on tonoplast, endoplasmic reticulum, and endosome or trans‐Golgi network/Golgi, respectively, in Arabidopsis.

The differential response displayed by transgenic rice plants expressing HtNHX1 or HtNHX2 would suggest their dissimilar cellular localization. Therefore, we used rice protoplasts expressing *HtNHX1‐GFP* or *HtNHX2‐GFP* encoding chimeras and several Arabidopsis markers to detect their localization. HtNHX1‐GFP, but not HtNHX2‐GFP fluorescence colocalized with the RFP signal of the tonoplast marker AtTPK1 (Figure 10a). Either HtNHX1‐GFP or HtNHX2‐GFP fluorescence was largely separated from RFP signal of the endoplasmic reticulum (ER) marker AtHDEL (Figure 10b).

It has been addressed that the type‐I NHX members including AtNHX1‐AtNHX4 are located mainly at the vacuolar membranes while the type‐II NHX members including AtNHX5, AtNHX6, LeNHX2, HvNHX4 and OsNHX5 are at endosome membranes (Bassil *et al*., 2012). We aligned sequences of HtNHX1 and HtNHX2 with those of known plant NHXs and found a patch of conserved eight amino acids (S/T,P/F,G,X,S/T,Ø,X,V) in type‐I NHXs is missed in the sequences of type‐II NHXs (Figure S1C). Interestingly, this eight amino acids motif is also missed in HtNHX2 (Figure S1C). To gain insight on the effect of these eight amino acids on the cellular localization of type‐I NHX members, we generated the HtNHX1del‐GFP encoding chimera and expressed it in rice protoplasts. Interestingly, the mutated HtNHX1 was separated from AtTPK1 location (Figure 10a), but largely overlapped with the ER marker (AtHDEL) (Figure 10b). The results suggested that the eight amino acids patch was important for targeting HtNHX1 to the vacuolar membrane.

In the rice protoplasts, HtNHX2 localized similar to AtNHX5 and partially overlapped with AtSYP61 and AtScamp (Figure 10c), indicating that HtNHX2 was largely distributed at endosomal membranes.

## Discussion

Plant NHX‐type antiporters play important roles in salt tolerance and the maintenance of cellular K^+^ and Na^+^ homoeostasis and the regulation of intracellular pH. In this study, we cloned two highly identical salt‐stress up‐regulated NHX orthologues genes, *HtNHX1* and *HtNHX2* from *H. tuberosus* (Figures 1 and S1). *HtNHX1* and *HtNHX2* share identical 5′‐UTR, 3′‐UTR and coding sequences, except for a fragment of consecutive 342 bp in *HtNHX1* which is absent in *HtNHX2*. Whether the two genes were alternatively spliced or evolutionary independently generated is not clear. According to their sequences, both genes are predicted to belong to the type‐I family and to locate at the vacuole (Figure S1B). The ability of HtNHX1 but not HtNHX2 to confer resistance to hygromycin in yeast and Arabidopsis (Figure S5) and different cellular localization in rice (Figure 10) would suggest functional differences between the two isoforms. Notably, the role of HtNHX2 in conferring tolerance to nutrient deficiency, in addition to conferring tolerance to salinity, provides a novel insight on NHX functions (Figures 5, 6, 7, 8, S3 and S4).

### The contribution of HtNHX1 and HtNHX2 to salt tolerance in rice depends on the K^+^‐supply levels

We observed that HtNHX1 or HtNHX2 improved rice tolerance to salt stress (comparison of Figures 2b,e,f and 3b with Figure 2a,c,d; Figure 4a,c), but they did not have significant influence on growth and uptake of K^+^ and Na^+^ at normal growth conditions (Figures 2a,c,d, 3a and 4a,c). At salinized conditions, HtNHX1 increased rice K^+^ and Na^+^ accumulation, that is keeping the same ratio of K^+^/Na^+^ as that in WT (Figure 3d). This indicated that HtNHX1 indistinguishably transported K^+^ and Na^+^, which is similar to that of AtNHX1 and AtNHX2 in Arabidopsis (Barragán *et al*., 2012; Bassil *et al*., 2011b). In addition, the K^+^ and Na^+^ contents of plant expressing *HtNHX1* were higher in the roots (about 40%–50%) and the shoots (about 10%–25%) than in WT plants (Figure 3b,c), supporting the role of HtNHX1 as type‐I NHXs (Figure 10a) in sequestering Na^+^(K^+^) into the vacuoles (Barragán *et al*., 2012; Bassil *et al*., 2011b). Interestingly, the accumulation and distribution of K^+^ and Na^+^ in plants expressing *HtNHX2* differed from that seen in *HtNHX1* plants, as *HtNHX2* plants accumulated relatively more Na^+^ and K^+^ (Figure 3c). These results were in agreement with the localization of HtNHX2 to intracellular compartments other than the vacuole (Figure 10a).

Endosomal trafficking and the vesicle fusion to the vacuole are important components of the response of plant cells to salinity. Transporters playing roles in cation sequestration, such as NHX1 (Apse *et al*., 1999) and H^+^‐pumps (Gaxiola *et al*., 2001), depend on vesicular trafficking for their delivery to the tonoplast. Thus, the expression of endosomal NHXs may influence protein trafficking from the Golgi/TGN to the vacuoles (Bassil *et al*., 2011), necessary for the response to high salinity. The up‐regulation of endosomal NHXs, such as AtNHX5 in response to salt stress, but not to osmotic shock, supported the role of endosomal AtNHXs (Bassil *et al*., 2011a; Yokoi *et al*., 2002). In addition, salinity induced bulk endocytosis, promoted the rapid increase in vacuolar volume and the accumulation of sodium into the vacuole (Baral *et al*., 2015; Hamaji *et al*., 2009; Leshem *et al*., 2006; Mimura *et al*., 2003). These results suggest that the overexpression of endosomal NHXs, such as HtNHX2, contributed to salt tolerance through several mechanisms: increasing vesicle fusion to the vacuole, contributing to the accumulation of Na^+^ and by increasing the trafficking of transporters that could explain the improved nutrient uptake in the transgenic plants.

HtNHX1 and HtNHX2 rescued the salt sensitivity of the Arabidopsis *Atnhx5 nhx6* double mutant at the same extent (Figure 9a,b), but HtNHX1 was more effective than HtNHX2 in conferring tolerance of Arabidopsis to external hygromycin (Figure S5). It has been shown that AtNHX5 and AtNHX6 located at endosomes, TGN and prevacuolar compartments and *nhx5 nhx6* double knockouts displayed abnormal vesicular trafficking and sensitivity to salinity (Bassil *et al*., 2011a). In rice, HtNHX2 showed similar cellular localization as AtNHX5 (Figure 10c); thus, it is plausible that HtNHX2 may play a similar role as AtNHX5 in the regulation of endosomal ion and pH homoeostasis. Nevertheless, these results should be taken with some caution as both *HtNHX1* and *HtNHX2* were expressed under the control of a constitutive promoter (ubiquitin) and abnormal localization due to unregulated expression cannot be ruled out.

### The expression of *HtNHX2*, but not *HtNHX1*, improved rice growth and grain yield

Potassium is a plant essential nutrient, and vacuolar K^+^ plays roles in the regulation of cellular volume and tissue expansion. Although plants grown under different K^+^ supply can change their vacuolar K^+^ contents, cytosolic K^+^ is maintained at a relative constant level of around 60–100 mm (Cuin *et al*., 2003; Walker *et al*., 1996). The activity of NHXs plays a significant role in the transport of K^+^ into the vacuole (Bassil *et al*., 2012). The overexpression of *AtNHX1* in tomato led to increased vacuolar K^+^ accumulation (Zhang and Blumwald, 2001) and higher tissue K^+^ contents. In the knockout *Atnhx1*, Apse *et al*. (2003) reported a reduction in K^+^/H^+^ and Na^+^/H^+^ exchange and reduced cell expansion. Bassil *et al*. (2011b) observed lower vacuole pH and K^+^ concentration in the *nhx1 nhx2* mutant, further supporting the role of AtNHX1 and AtNHX2 in driving the uptake of K^+^ into the vacuole.

The expression of *HtNHX2* improved plant growth and grain yield at low K^+^ concentrations (Figures 5, S3), and the tissue K^+^ contents of the transgenic plants were higher than the WT and *HtNHX1‐*expressing plants. Notably, HtNHX2 localization appeared to be similar to that of AtNHX5 (Figure 10), that is intracellular vesicles, TGN. (Bassil *et al*., 2011a), thus associated with vesicular trafficking and storage protein sorting (Reguera *et al*., 2014). In yeast, *nhx1* mutants have been shown to have impaired vacuolar biogenesis and protein sorting (Qiu and Fratti, 2010). In tomato, LeNHX2 colocalized with prevacuolar and Golgi markers and appeared to be more selective to K^+^ than Na^+^ (Venema *et al*., 2003). Interestingly, HtNHX2 lacks a continuous 114 amino acids stretch at predicted six to eight transmembrane domains of HtNHX1 (Figure S1D). This stretch also comprises a segment of eight amino acids that is absent in type‐II NHXs (Figure S1C). Although the occurrence of a shorter HtNHX2 isoform retaining its transport function has not been reported before, shorter AtNHX1 isoforms have been reported (Xu *et al*., 2010). Using DNA shuffling mutagenesis, Xu *et al*. (2010) generated a 296 amino acids deleted AtNHX1, AtNHXS1, in which, several transmembrane domains and the C‐terminus hydrophilic tail containing the CaM‐binding domain (Yamaguchi *et al*., 2003, 2005) were deleted. Interestingly, such large fragment deletion did not alter its vacuolar localization but increased the Na^+^/K^+^ selectivity and Na^+^/H^+^ exchange activity, enhancing the NaCl tolerance of yeast expressing AtNHXS1 (Xu *et al*., 2010). Notably, plants expressing the endosomal HtNHX2 accumulated more K^+^ and Na^+^ than the transgenic rice expressing the vacuolar HtNHX1. Although it is possible to speculate that the deletion in HtNHX2 resulted in increased transport activity of the antiporter, a detailed structure/function analysis is needed to assess this point.

The increased tolerance to nutrient deficiency displayed by transgenic rice plants expressing *HtNHX2* was associated with increased K^+^ contents and enhanced N assimilation as seen by the increase in ^15^N translocation from root to shoot (Figure S4) and the amounts of N, P and K^+^ in the straw of plants grown in infertile soil (Figure 8a,b). K^+^ is the major accompanying ion for the translocation of NO_3_^−^ and sucrose in plants (Drew and Saker, 1984; Drew *et al*., 1990). About 40%–90% of root acquired K^+^ could be retranslocated from the shoot via the phloem and recycled through the roots (Lu *et al*., 2005; Peuke, 2010), and K^+^ recycling in plants can act as an important signal for feedback control of nutrient uptake (Drew *et al*., 1990; Engels and Marschner, 1992). It could be speculated that the expression of *HtNHX2* improved the synthesis/delivery of the transporters of N, P and K^+^ to plasma membranes by improving vesicular trafficking and/or protein targeting (Reguera *et al*., 2014).

The functions of NHX‐type transporters have been extensively studied (Bassil *et al*., 2012 and references therein), and the effects of the overexpression of NHXs on salt tolerance have been shown in different crop species (Bassil and Blumwald, 2014). Here, we show that although the expression of HtNHX1 conferred salt tolerance, only the expression of HtNHX2, a shorter isoform ot the known type‐II NHXs, conferred salt tolerance under nutrient stress conditions.

## Experimental procedures

### Plant materials and growth conditions

The tubers of *H. tuberosus* genotype NY‐1 after dormancy were germinated on moist sand in an incubator. Uniformly germinated slices of the buds were selected, sown in sands and then transplanted into 1/2 Hoagland nutrient solution in the greenhouse as described previously (Li *et al*., 2014). After emergence of the fourth leaf, the seedlings were treated with 50, 100, 200 and 300 mm NaCl for 24 h or with 100 mM NaCl for 12 h before the sampling for analysis of *HtNHX1* and *HtNHX2* expression. Each treatment was replicated three times.

*Arabidopsis thaliana* (ecotype Columbia (Col‐0) wild‐type (WT) and *nhx5 nhx6* double knockout mutants (Bassil *et al*., 2011b) were provided by Professor Eduardo Blumwald at University of California (Davis, California). For the salt and hygromycin tolerance analysis in agar medium, the seeds were germinated and grown by following the method described by Bassil *et al*. (2011b). For the salt tolerance analysis in the pot filled with peat culture medium, the seedlings of uniform size after 2 weeks growth in a plate were selected and transplanted to the nutrient‐enriched medium in a growth chamber at 23 °C with a 14 : 10 h L : D photoperiod. For salt treatment, NaCl was added to final concentrations as indicated in the figure legends.

The rice hypotonic culture and soil pot culture were basically followed the protocols previously described by Li *et al*. (2014) and Yang *et al*. (2014). Briefly, for hypotonic culture, 2‐week‐old seedlings of similar size were transferred to IRRI nutrient solution with specific treatment (indicated in figure legends) for additional 3 weeks. The nutrient concentrations (mm) of IRRI solution are as follows: N 2.5, P 0.3, K 1, Ca 1, Mg 1, Si 0.5, Fe 0.02, B 0.02, Mn 0.009, Zn 0.00077, Cu 0.00032, Mo 0.00039. For salt‐stressed pot culture, 2‐week‐old seedlings were acclimated to the soil containing 0.1% (1‐g/kg soil) NaCl for 1 week before being transplanted to the pot filled with 40 kg of soil which had been fertilized and mixture with indicated extra amount of salts (0, 1.3 and 1.8‐g/kg soil, respectively) for 2 weeks. For either K^+^ deficient or N + P deficient soil culture, 4‐week‐old seedlings were transplanted to the respective isolated paddy field and no extra fertilizer was added during the growth season. The soil properties used for the plant tolerance to salt stress, K^+^ deficiency or N+P deficiency were described in Table S2.

### Cloning of *HtNHX1* and *HtNHX2* sequences

Both cDNAs of *HtNHX1* and *HtNHX2* were reversely transcribed from the RNA extracted from the leaves of *NY‐1*. Degenerate primers (P1, P2) designed for cloning the Na^+^/H^+^ antiporters (Table S1) were based on alignment of the sequences of yeast, microbes, moss and plant Na^+^/H^+^ antiporters. The amplified fragments were ligated into the pEasy‐Blunt vector (Transgene, China). Based on the sequences of this RT‐PCR fragment, rapid‐amplification of cDNA ends (RACE) PCR was carried out using SMARTer RACE cDNA amplification kit (Clontech, China) to obtain the full‐length cDNA sequences according to the instruction. First strand of 5′‐ and 3′‐RACE‐ready cDNA was synthesized according to the kit manual with total RNA. The 3′ and 5′ RACE‐PCRs were carried out with each pair of gene‐specific and universal primers using the first strand cDNA as the template. The 3′ nested PCR was performed with the nested gene‐specific primer and nested universal primer A (provided with the kit) using diluted 3′ RACE‐PCR product as template because the level of background in the 3′‐RACES reaction was too high. The primers used in RACE‐PCR were designed based on the partial DNA sequence: P3: (for 5′ RACE‐PCR), P4: (for 5′ nested RACE‐PCR), P5: (for 3′ RACE‐PCR), and P6: (for 3′ nested RACE‐PCR). The PCR product was purified and cloned into pEasy‐Blunt vector and sequenced. By aligning the sequences of the 5′‐end 3′‐end PCR products and the previous partial DNA sequence, the full‐length cDNA sequence of *HtNHX1* and *HtNHX2* was deduced and obtained by RT‐PCR with KOD‐PLUS (TOYOBO, Japan) using the specific primers: P7 and P8 (Table S1).

### *HtNHX1* and *HtNHX2* transcripts analysis

Total RNA was extracted from root or shoot of *H. tuberosus* genotype NY‐1 subjected to different salt stresses. Real‐time qRT‐PCR analysis of *HtNHX1* was performed as described by Li *et al*. (2014). Semi‐quantitative RT‐PCR analysis for *HtNHX2* was performed according to the protocol described previously (Tsuchiya *et al*., 2004; Yan *et al*., 2011). The target genes and actin standards in 1 : 10, 1 : 100 and 1 : 1000 dilutions were always present in the experiments. All the primers are listed in Table S1. All the PCR products were checked by electrophoresis and sequenced to confirm their identity.

### Generating the transgenic rice expressing either *HtNHX1* or *HtNHX2*

The open reading frames of *HtNXH1* and *HtNXH2* were amplified by gene‐specific primers (see Table S1). The fragment was treated with restriction enzymes and inserted into pTCK303 expression vectors under the control of the Ubi‐promoter. The final constructed vector was sequenced before transformation. The gene transformation into rice (*Oryza sativa* L. cv. Nipponbare) was performed by Agrobacterium‐mediated cocultivation method (Upadhyaya *et al*., 2010; Li *et al*., 2014). Total 13 lines of *HtNHX1* and 17 lines of *HtNHX2* expressed homozygotes were initially obtained from T1 generation. By Southern blot analysis, each five lines of *HtNHX1* or *HtNHX2* expressing rice plants with only one copy of the gene insertion (Figure S2A) were selected and propagated for getting the seeds of T2‐T4 generations. The exact integration sites of *HtNHX1* or *NtNHX2* in the rice genome for *HtNHX1.1‐HtNHX1.2* and *HtNHX2.1‐HtNHX2.2* lines were sequenced (Figure S2B), and their T3 generation was used for further experiments.

### Expression of *HtNH1* or *HtNHX2* gene in Arabidopsis *nhx5 nhx6* double mutant

The *HtNHX1‐* or *HtNHX2‐*coding sequence was amplified and built into the BamHI/SacI sites of the pTCK303 vector driven by the ubiquitin promoter. The resulting constructs were introduced into *nhx5‐2 nhx6‐2* for the functional complementation by *Agrobacterium tumefaciens* strain EHA105 using the floral dipping method (Clough and Bent, 1998).

### Southern blot and TAIL‐PCR analysis

The independent transgenic lines with *HtNHX1* or *HtNHX2* expression lines, *NHX1‐1*,* NHX1‐2* and *NHX2‐1*,* NHX2‐2*, were determined by Southern blot analysis following the procedures described previously (Yan *et al*., 2011). A TAIL‐PCR procedure was performed as described previously (Liu *et al*., 1995). The PCR products were purified and then cloned into pMD‐19 T vector (TaKaRa Biotechnology Co., Ltd., Dalian, China) for sequencing. Sequence identity was determined by blasting against the NCBI database. All of the primers used for TAIL‐PCR and procedures are listed in Table S1.

### HtNHX1 and HtNHX2 subcellular localization assay

Rice protoplasts were isolated according to Yang *et al*. (2014), C‐terminal or N‐terminal GFP fused HtNHX1 or HtNHX2 was constructed in the vector and transiently expressed in the rice protoplasts and detected by laser scanning microscope (LSM410; Carl Zeiss) as described by Yang *et al*. (2014). The separation of the membrane fractions of BY2 cells was conducted according to the bio‐protocol of Membrane Preparation, Sucrose Density Gradients and Two‐phase Separation Fractionation from 5‐day‐old Arabidopsis seedlings (http://www.bio-protocol.org/e1014) by Yang *et al*. (2013).

### Determination total Na^+^, K^+^, N and P content in rice

All the samples were dried at 105 °C for 30 min and then kept at 70 °C in an oven for 72 h before weighing. After grinding, the samples were extracted by 2 m HCl for 2 days before measuring K^+^ and Na^+^ by a flame emission spectrometry. For N and P, samples were digested completely with 98% H_2_SO_4_–30% H_2_O_2_ and measured as described by Tang *et al*. (2012) and Ai *et al*. (2009).

### Statistical analysis

Data were analysed by ANOVA using the SPSS 10 program (SPSS). Different letters above columns in the figures indicate statistical differences (Probability at 0.05) between individual transgenic lines and wild‐type and/or between different treatments.

## Conflict of interest

The authors declare no conflict of interest.

## Supporting information


**Figure S1** Bioinformatic prediction of HtNHX1 and HtNHX2 structures.
**Figure S2** Isolation of single copy inserted rice lines of *HtNHX1* or *HtNHX2* expression.
**Figure S3** Effects of overexpression of *HtNHX1* and *HtNHX2* on grain yield, harvest index, and NPK concentrations in the rice grown in low K supplied soil.
**Figure S4** Effects of *HtNHX1* and *HtNHX2* expression on ammonium N acquisition and distribution under low N and K supply condition.
**Figure S5** HtNHX1, but not HtNHX2, functioned in enhancing tolerance to external hygromycin in both nhx1 mutated yeast cells (A) and Arabidopsis (B).
**Table S1** The primers used in the experiments.
**Table S2** The properties of the soil used in the experiments.
