# Supplementary material for: Two NHX‐type transporters from Helianthus tuberosus improve the tolerance of rice to salinity and nutrient deficiency stress
Source: Plant Biotechnol J. 2017 Sep 21;16(1):310–21. doi: 10.1111/pbi.12773 (PMC5785360; doi:10.1111/pbi.12773)
Supplement: Supplementary file 1 — Figure S1 Bioinformatic prediction of HtNHX1 and HtNHX2 structures. Figure S2 Isolation of single copy inserted rice lines of HtNHX1 or HtNHX2 expression. Figure S3 Effects of overexpression of HtNHX1 and HtNHX2 on grain yield, harvest index, and NPK concentrations in the rice grown in low K supplied soil. Figure S4 Effects of HtNHX1 and HtNHX2 expression on ammonium N acquisition and distribution under low N and K supply condition. Figure S5 HtNHX1, but not HtNHX2, functioned in enhancing tolerance to external hygromycin in both nhx1 mutated yeast cells (A) and Arabidopsis (B). Table S1 The primers used in the experiments. Table S2 The properties of the soil used in the experiments. [file PBI-16-310-s001.pptx]

## Slide 1
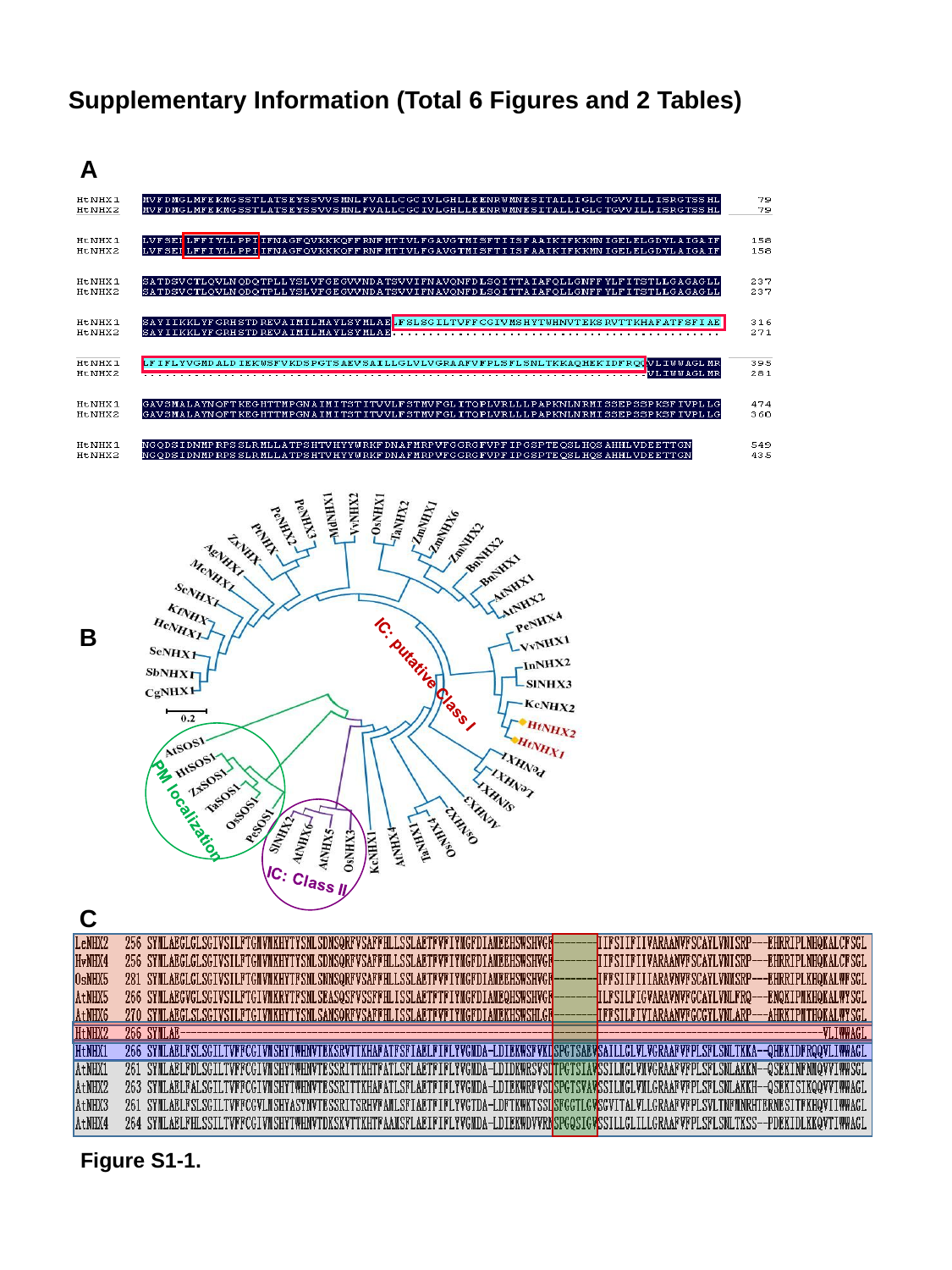

Supplementary Information (Total 6 Figures and 2 Tables)
A
B
C
Figure S1-1.

## Slide 2
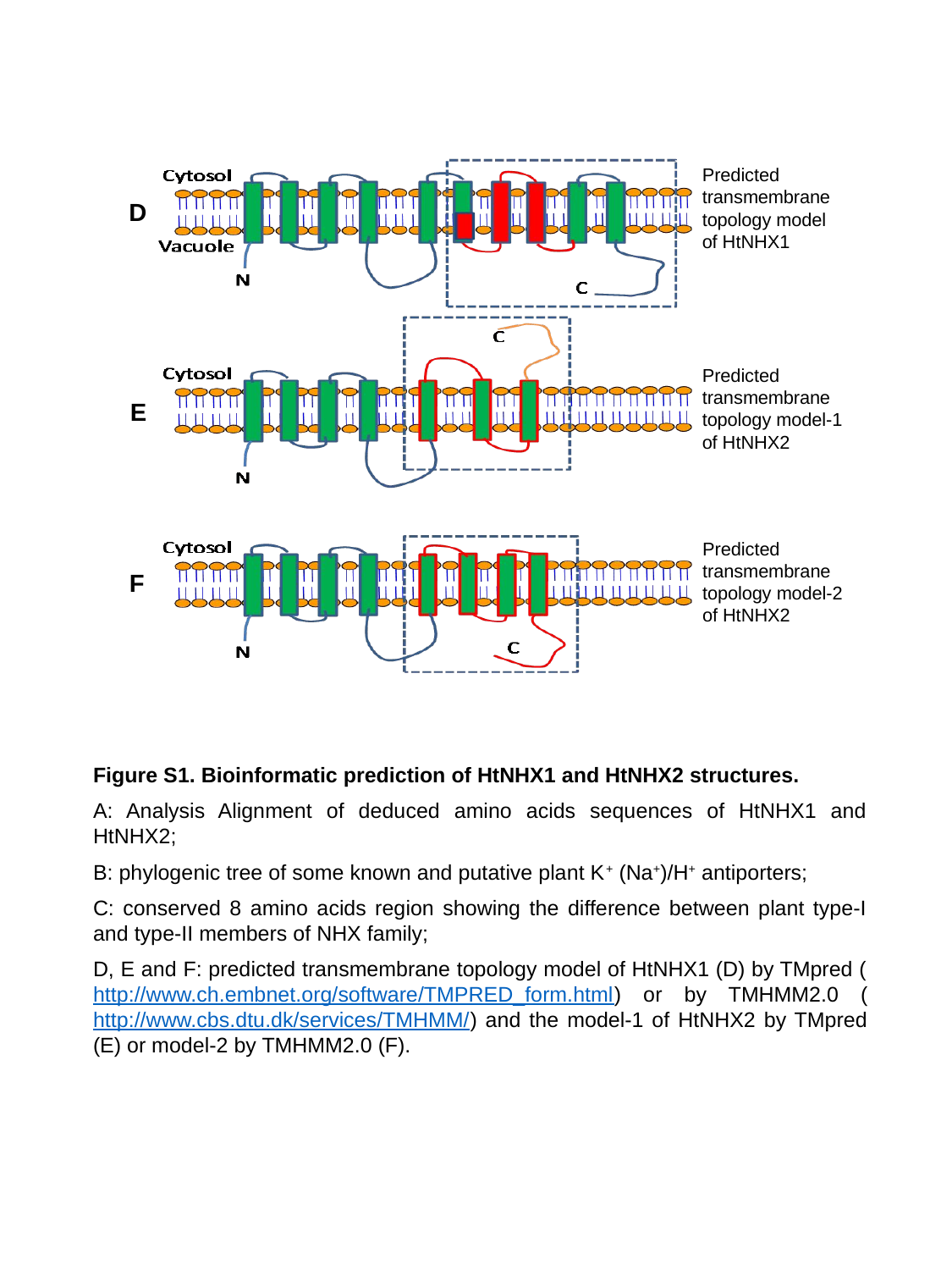

Predicted transmembrane topology model of HtNHX1
D
Predicted transmembrane topology model-1 of HtNHX2
E
Predicted transmembrane topology model-2 of HtNHX2
F
Figure S1. Bioinformatic prediction of HtNHX1 and HtNHX2 structures.
A: Analysis Alignment of deduced amino acids sequences of HtNHX1 and HtNHX2;
B: phylogenic tree of some known and putative plant K+ (Na+)/H+ antiporters;
C: conserved 8 amino acids region showing the difference between plant type-I and type-II members of NHX family;
D, E and F: predicted transmembrane topology model of HtNHX1 (D) by TMpred (http://www.ch.embnet.org/software/TMPRED_form.html) or by TMHMM2.0 (http://www.cbs.dtu.dk/services/TMHMM/) and the model-1 of HtNHX2 by TMpred (E) or model-2 by TMHMM2.0 (F).

## Slide 3
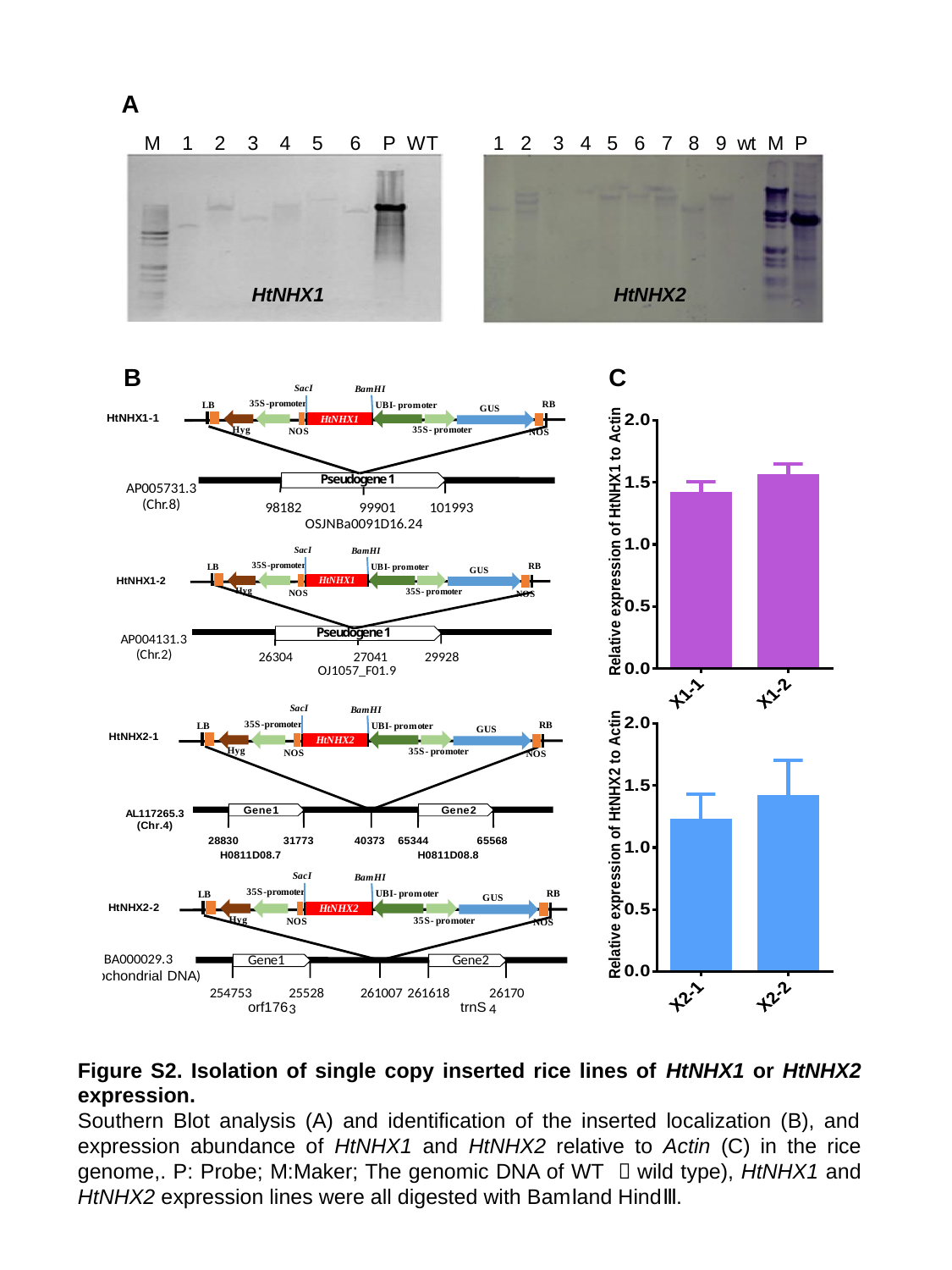

A
B
C
Figure S2. Isolation of single copy inserted rice lines of HtNHX1 or HtNHX2 expression.
Southern Blot analysis (A) and identification of the inserted localization (B), and expression abundance of HtNHX1 and HtNHX2 relative to Actin (C) in the rice genome,. P: Probe; M:Maker; The genomic DNA of WT （wild type), HtNHX1 and HtNHX2 expression lines were all digested with BamⅠand HindⅢ.

## Slide 4
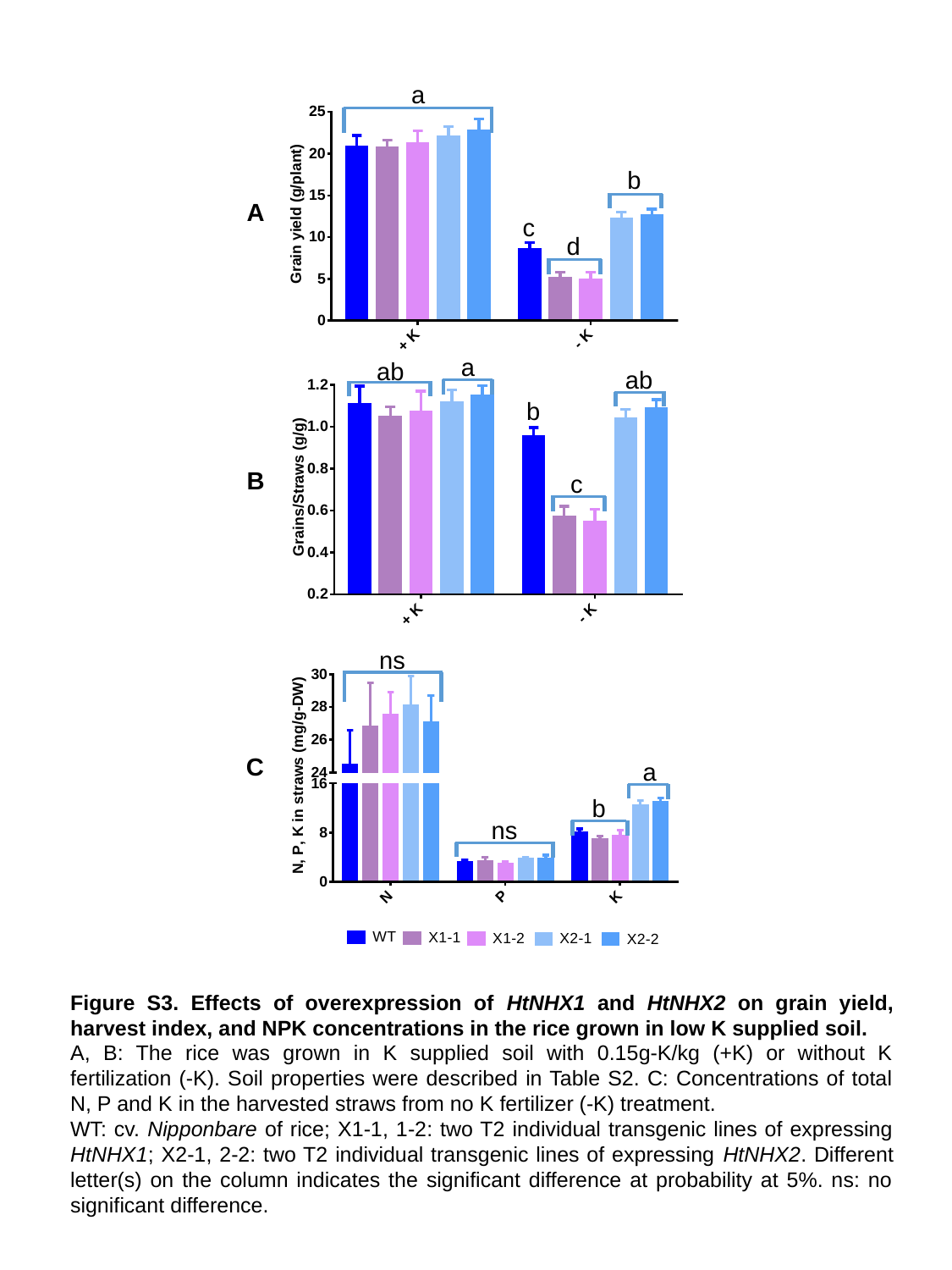

a
b
A
c
d
a
ab
ab
b
B
c
ns
C
a
b
ns
Figure S3. Effects of overexpression of HtNHX1 and HtNHX2 on grain yield, harvest index, and NPK concentrations in the rice grown in low K supplied soil.
A, B: The rice was grown in K supplied soil with 0.15g-K/kg (+K) or without K fertilization (-K). Soil properties were described in Table S2. C: Concentrations of total N, P and K in the harvested straws from no K fertilizer (-K) treatment.
WT: cv. Nipponbare of rice; X1-1, 1-2: two T2 individual transgenic lines of expressing HtNHX1; X2-1, 2-2: two T2 individual transgenic lines of expressing HtNHX2. Different letter(s) on the column indicates the significant difference at probability at 5%. ns: no significant difference.

## Slide 5
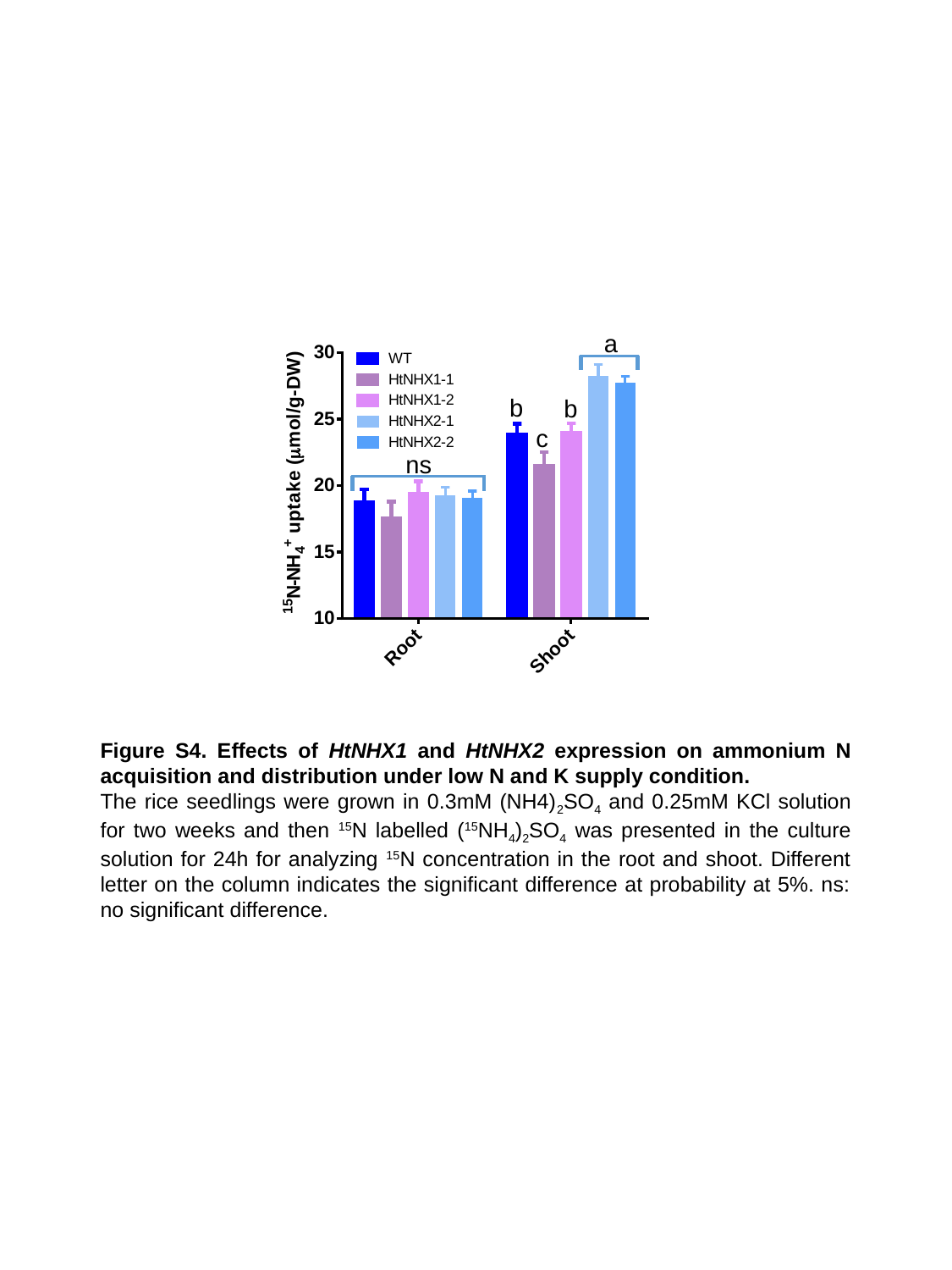

a
b
b
c
ns
Figure S4. Effects of HtNHX1 and HtNHX2 expression on ammonium N acquisition and distribution under low N and K supply condition.
The rice seedlings were grown in 0.3mM (NH4)2SO4 and 0.25mM KCl solution for two weeks and then 15N labelled (15NH4)2SO4 was presented in the culture solution for 24h for analyzing 15N concentration in the root and shoot. Different letter on the column indicates the significant difference at probability at 5%. ns: no significant difference.

## Slide 6
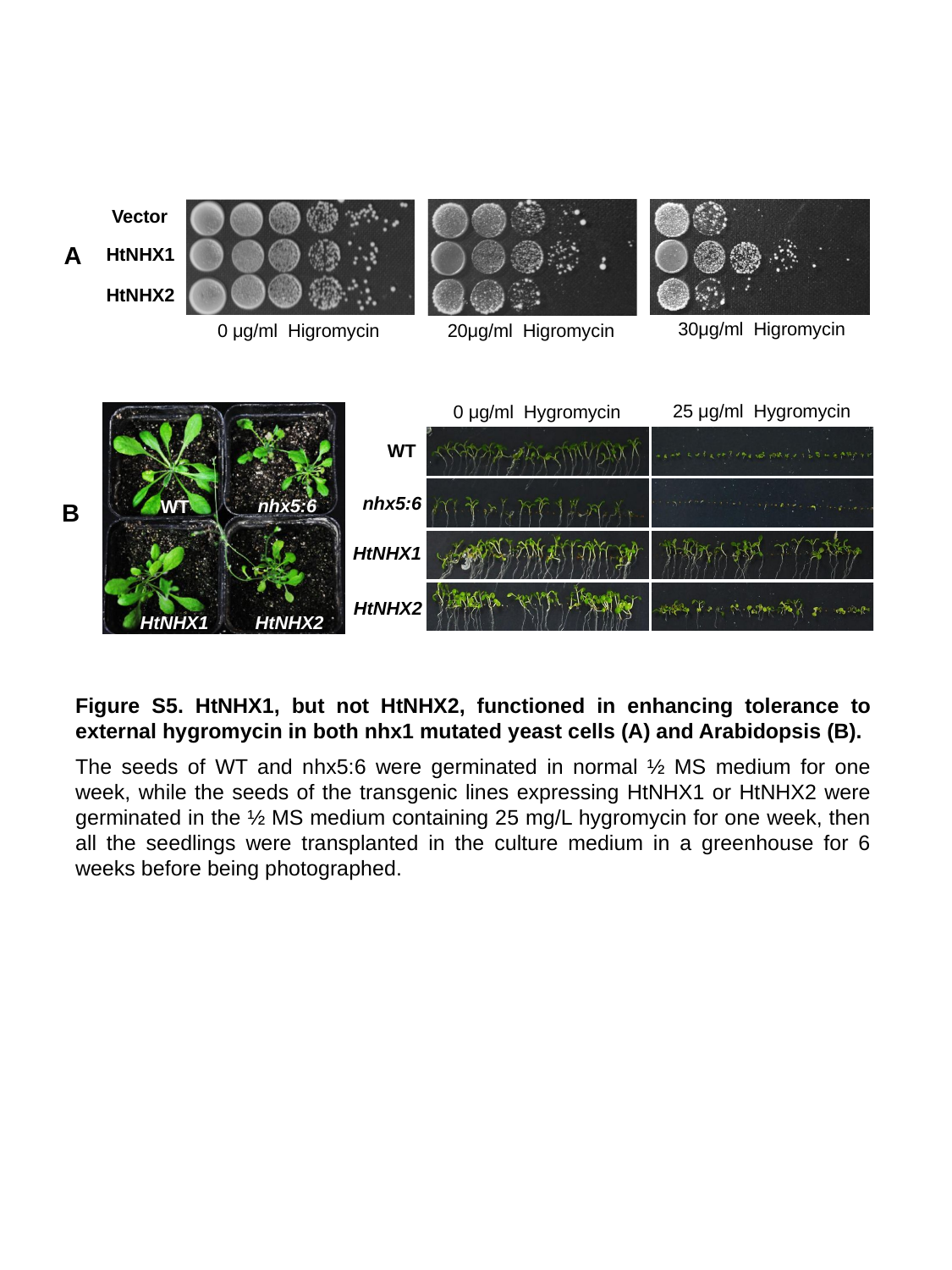

Vector
HtNHX1
HtNHX2
30μg/ml Higromycin
0 μg/ml Higromycin
20μg/ml Higromycin
A
nhx5:6
WT
HtNHX1
HtNHX2
B
25 μg/ml Hygromycin
0 μg/ml Hygromycin
WT
nhx5:6
HtNHX1
HtNHX2
Figure S5. HtNHX1, but not HtNHX2, functioned in enhancing tolerance to external hygromycin in both nhx1 mutated yeast cells (A) and Arabidopsis (B).
The seeds of WT and nhx5:6 were germinated in normal ½ MS medium for one week, while the seeds of the transgenic lines expressing HtNHX1 or HtNHX2 were germinated in the ½ MS medium containing 25 mg/L hygromycin for one week, then all the seedlings were transplanted in the culture medium in a greenhouse for 6 weeks before being photographed.

## Slide 7
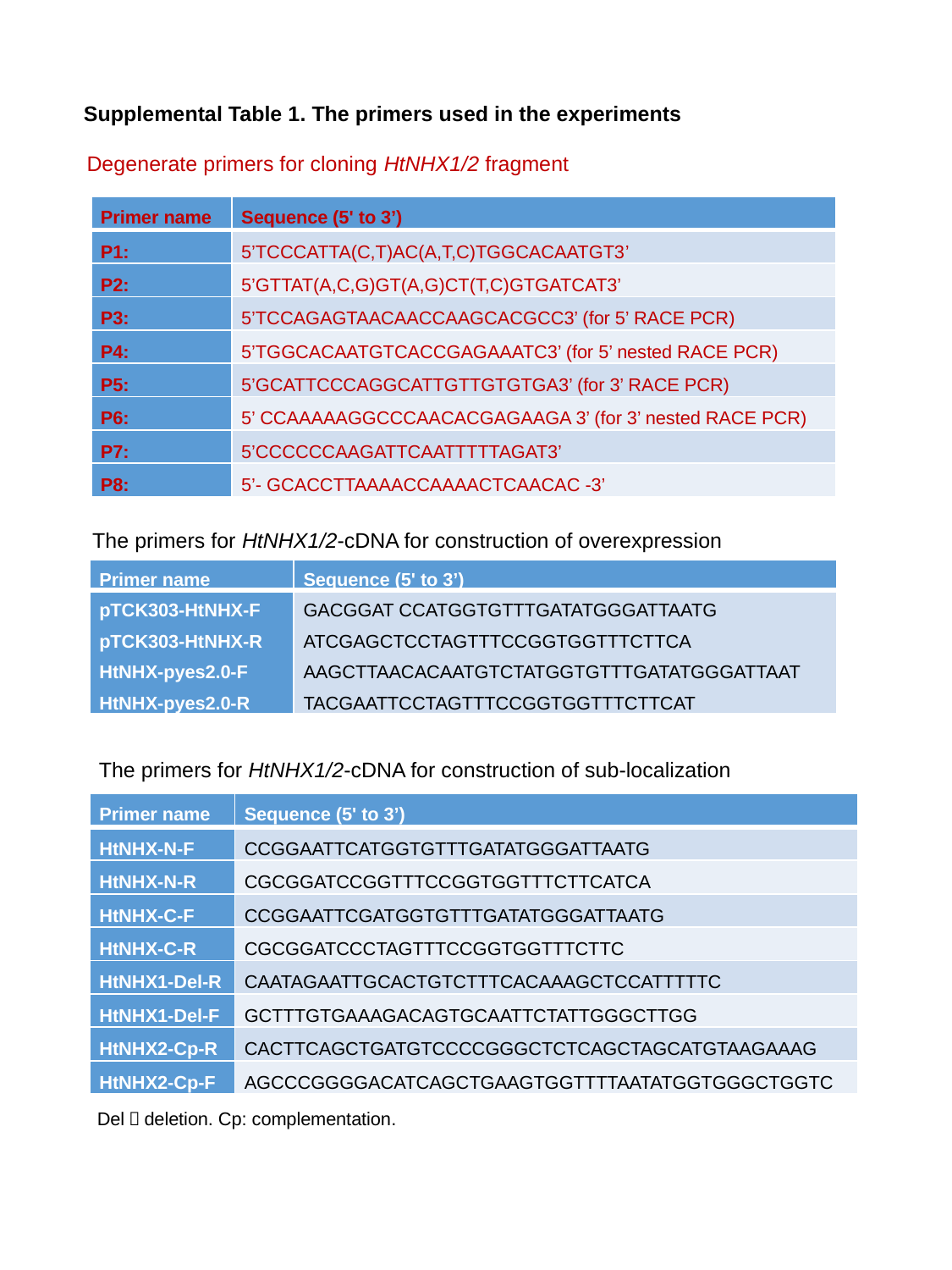

# Supplemental Table 1. The primers used in the experiments
Degenerate primers for cloning HtNHX1/2 fragment
| Primer name | Sequence (5' to 3’) |
| --- | --- |
| P1: | 5’TCCCATTA(C,T)AC(A,T,C)TGGCACAATGT3’ |
| P2: | 5’GTTAT(A,C,G)GT(A,G)CT(T,C)GTGATCAT3’ |
| P3: | 5’TCCAGAGTAACAACCAAGCACGCC3’ (for 5’ RACE PCR) |
| P4: | 5’TGGCACAATGTCACCGAGAAATC3’ (for 5’ nested RACE PCR) |
| P5: | 5’GCATTCCCAGGCATTGTTGTGTGA3’ (for 3’ RACE PCR) |
| P6: | 5’ CCAAAAAGGCCCAACACGAGAAGA 3’ (for 3’ nested RACE PCR) |
| P7: | 5’CCCCCCAAGATTCAATTTTTAGAT3’ |
| P8: | 5’- GCACCTTAAAACCAAAACTCAACAC -3’ |
The primers for HtNHX1/2-cDNA for construction of overexpression
| Primer name | Sequence (5' to 3’) |
| --- | --- |
| pTCK303-HtNHX-F pTCK303-HtNHX-R HtNHX-pyes2.0-F HtNHX-pyes2.0-R | GACGGAT CCATGGTGTTTGATATGGGATTAATG ATCGAGCTCCTAGTTTCCGGTGGTTTCTTCA AAGCTTAACACAATGTCTATGGTGTTTGATATGGGATTAAT TACGAATTCCTAGTTTCCGGTGGTTTCTTCAT |
The primers for HtNHX1/2-cDNA for construction of sub-localization
| Primer name | Sequence (5' to 3’) |
| --- | --- |
| HtNHX-N-F | CCGGAATTCATGGTGTTTGATATGGGATTAATG |
| HtNHX-N-R | CGCGGATCCGGTTTCCGGTGGTTTCTTCATCA |
| HtNHX-C-F | CCGGAATTCGATGGTGTTTGATATGGGATTAATG |
| HtNHX-C-R | CGCGGATCCCTAGTTTCCGGTGGTTTCTTC |
| HtNHX1-Del-R | CAATAGAATTGCACTGTCTTTCACAAAGCTCCATTTTTC |
| HtNHX1-Del-F | GCTTTGTGAAAGACAGTGCAATTCTATTGGGCTTGG |
| HtNHX2-Cp-R | CACTTCAGCTGATGTCCCCGGGCTCTCAGCTAGCATGTAAGAAAG |
| HtNHX2-Cp-F | AGCCCGGGGACATCAGCTGAAGTGGTTTTAATATGGTGGGCTGGTC |
Del：deletion. Cp: complementation.

## Slide 8
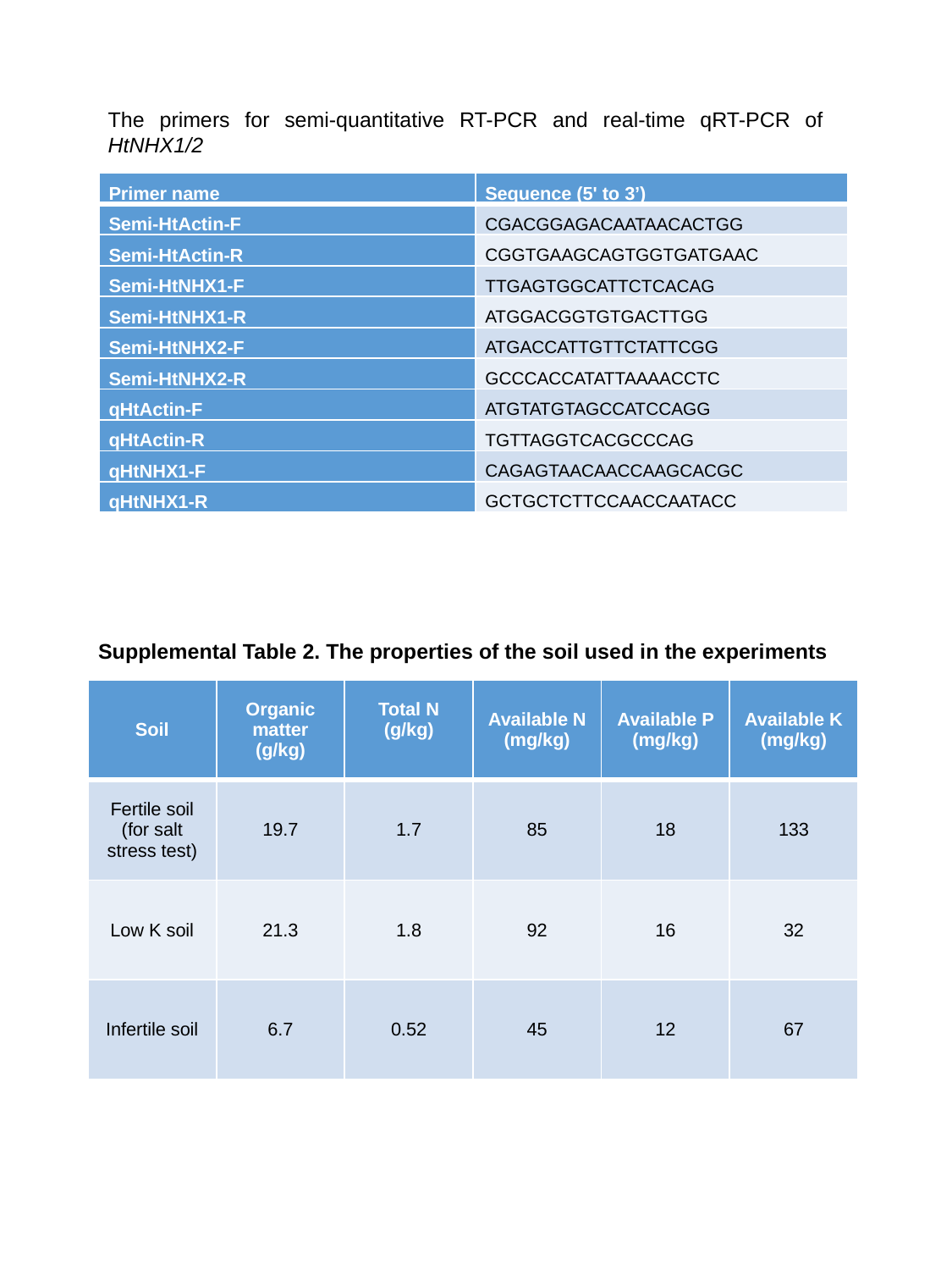

The primers for semi-quantitative RT-PCR and real-time qRT-PCR of HtNHX1/2
| Primer name | Sequence (5' to 3’) |
| --- | --- |
| Semi-HtActin-F | CGACGGAGACAATAACACTGG |
| Semi-HtActin-R | CGGTGAAGCAGTGGTGATGAAC |
| Semi-HtNHX1-F | TTGAGTGGCATTCTCACAG |
| Semi-HtNHX1-R | ATGGACGGTGTGACTTGG |
| Semi-HtNHX2-F | ATGACCATTGTTCTATTCGG |
| Semi-HtNHX2-R | GCCCACCATATTAAAACCTC |
| qHtActin-F | ATGTATGTAGCCATCCAGG |
| qHtActin-R | TGTTAGGTCACGCCCAG |
| qHtNHX1-F | CAGAGTAACAACCAAGCACGC |
| qHtNHX1-R | GCTGCTCTTCCAACCAATACC |
# Supplemental Table 2. The properties of the soil used in the experiments
| Soil | Organic matter (g/kg) | Total N (g/kg) | Available N (mg/kg) | Available P (mg/kg) | Available K (mg/kg) |
| --- | --- | --- | --- | --- | --- |
| Fertile soil (for salt stress test) | 19.7 | 1.7 | 85 | 18 | 133 |
| Low K soil | 21.3 | 1.8 | 92 | 16 | 32 |
| Infertile soil | 6.7 | 0.52 | 45 | 12 | 67 |
